# Supplementary material for: Structural basis of broad SARS-CoV-2 cross-neutralization by affinity-matured public antibodies
Source: Cell Rep Med. 2024 May 17;5(6):101577. doi: 10.1016/j.xcrm.2024.101577 (PMC11228396; doi:10.1016/j.xcrm.2024.101577)
Supplement: Document S1. Figures S1‒S4 and Tables S1‒S3 [file mmc1.pdf]

Cell Reports Medicine, Volume *5*

Supplemental information

Structural basis of broad SARS-CoV-2 cross-neutralization by affinity-matured public antibodies

Daniel J. Sheward, Pradeepa Pushparaj, Hrishikesh Das, Allison J. Greaney, Changil Kim, Sungyong Kim, Leo Hanke, Erik Hyllner, Robert Dyrdak, Jimin Lee, Xaquin Castro Dopico, Pia Dosenovic, Thomas P. Peacock, Gerald M. McInerney, Jan Albert, Martin Corcoran, Jesse D. Bloom, Ben Murrell, Gunilla B. Karlsson Hedestam, and B. Martin Hällberg

Cell Reports Medicine

Structural basis of broad SARS-CoV-2 cross-neutralization by affinity-matured public antibodies

Daniel J. Sheward, Pradeepa Pushparaj, Hrishikesh Das, Allison J. Greaney, Changil Kim, Sungyong Kim, Leo Hanke, Erik Hyllner, Robert Dyrdak, Jimin Lee, Xaquin Castro Dopico, Pia Dosenovic, Thomas P. Peacock, Gerald M. McInerney, Jan Albert, Martin Corcoran, Jesse D. Bloom, Ben Murrell, Gunilla B. Karlsson Hedestam, and B. Martin Hällberg

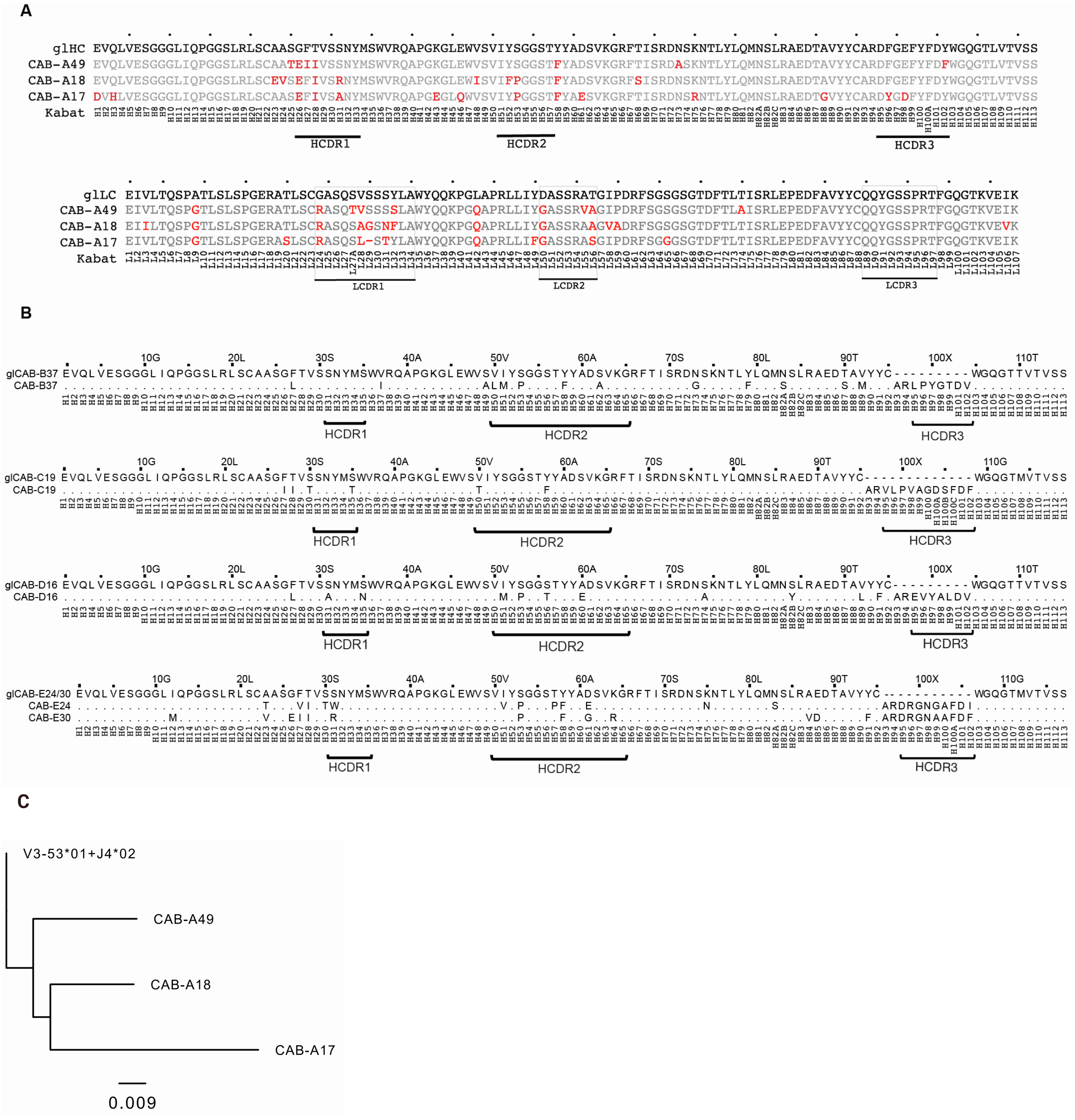

Figure S1. Alignment of sequences for the four lineages studied in this work. Related to Figure 1. **(A)** Alignment of heavy- and light-chain sequences of mAbs from lineage CAB-A. CDR borders and numbering are shown according to the Kabat scheme. Somatic hypermutations away from the germline (gl) are shown in red. **(B)** Alignment of heavy-chain sequences with their inferred germline for antibody lineages B-E. **(C)** A phylogeny of the CAB-A lineage, inferred from the heavy chain CAB-A17, CAB-A18, and CAB-A49 sequences, rooted on the inferred germline V and J genes.

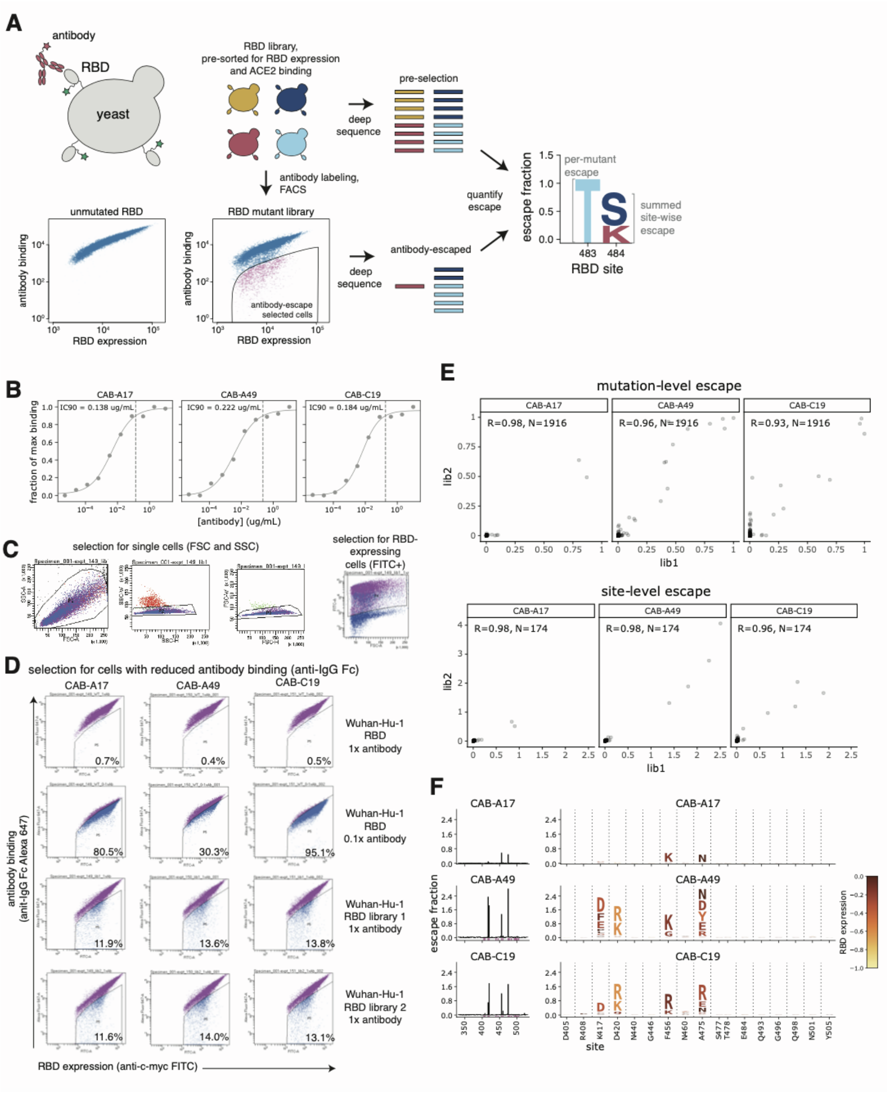

Figure S2. Complete mapping of mutations that escape antibody binding to the Wuhan-Hu-1 RBD. Related to Figures 1D-E. **(A)** A previously described deep mutational scanning system was used to map mutations that escape antibody binding.[27] A library of yeast containing nearly all possible mutations to the Wu-Hu-1 RBD was incubated with antibody and fluorescence-activated cell sorting (FACS) was used to enrich for cells expressing RBD (detected with a C-terminal MYC tag, green star) with reduced antibody binding, detected using an anti-human IgG Fc-gamma secondary antibody. Deep sequencing was used to quantify the frequency of each mutation in the pre-selection and antibody-escape cell populations. We calculated each mutation's "escape fraction," the fraction of cells expressing RBD with that mutation that fell in the antibody escape FACS bin (ranging from 0 to 1). The site-level escape metric is the sum of the escape fractions of all mutations at a site. Experimental and computational filters were used to remove RBD mutants that were misfolded or unable to bind the ACE2 receptor as described.[25,27] **(B)** Antibody binding to the yeast-displayed unmutated Wu-Hu-1 RBD was measured by flow cytometry and Wu-Hu-1 RBD libraries were incubated with antibody at the IC90 ("1x antibody") for antibody-escape selections. **(C)** Representative plots of nested FACS gating strategy used to select for RBD+ single cells. Samples were gated by SSC-A versus FSC-A, SSC-W versus SSC-H, and FSC-W versus FSC-H) that also express RBD (FITC-A vs. FSC-A). (D) FACS gating strategy to select cells expressing RBD mutants with reduced antibody binding (cells in blue). Selection gates were set to exclude cells expressing unmutated Wu-Hu-1 RBD stained with 1x antibody (top row), and to capture up to 95% of cells expressing unmutated Wu-Hu-1 RBD stained with 0.1x antibody (second row from top). The FACS scatter plots are shown for the two library replicates. SSC-A, side scatter-area; FSC-A, forward scatter-area; SSC-W, side scatter-width; SSC-H, side scatter-height; FSC-W, forward scatter-width; FSC-H, forward scatter height; FITC-A, fluorescein isothiocyanate-area. (E) Mutation (top)- and site (bottom)-level correlations of escape scores between two independent biological replicate libraries. (F) The antibody-escape maps as shown in Figure 1D, but with mutations colored according to effect on RBD expression, as previously measured.[29]

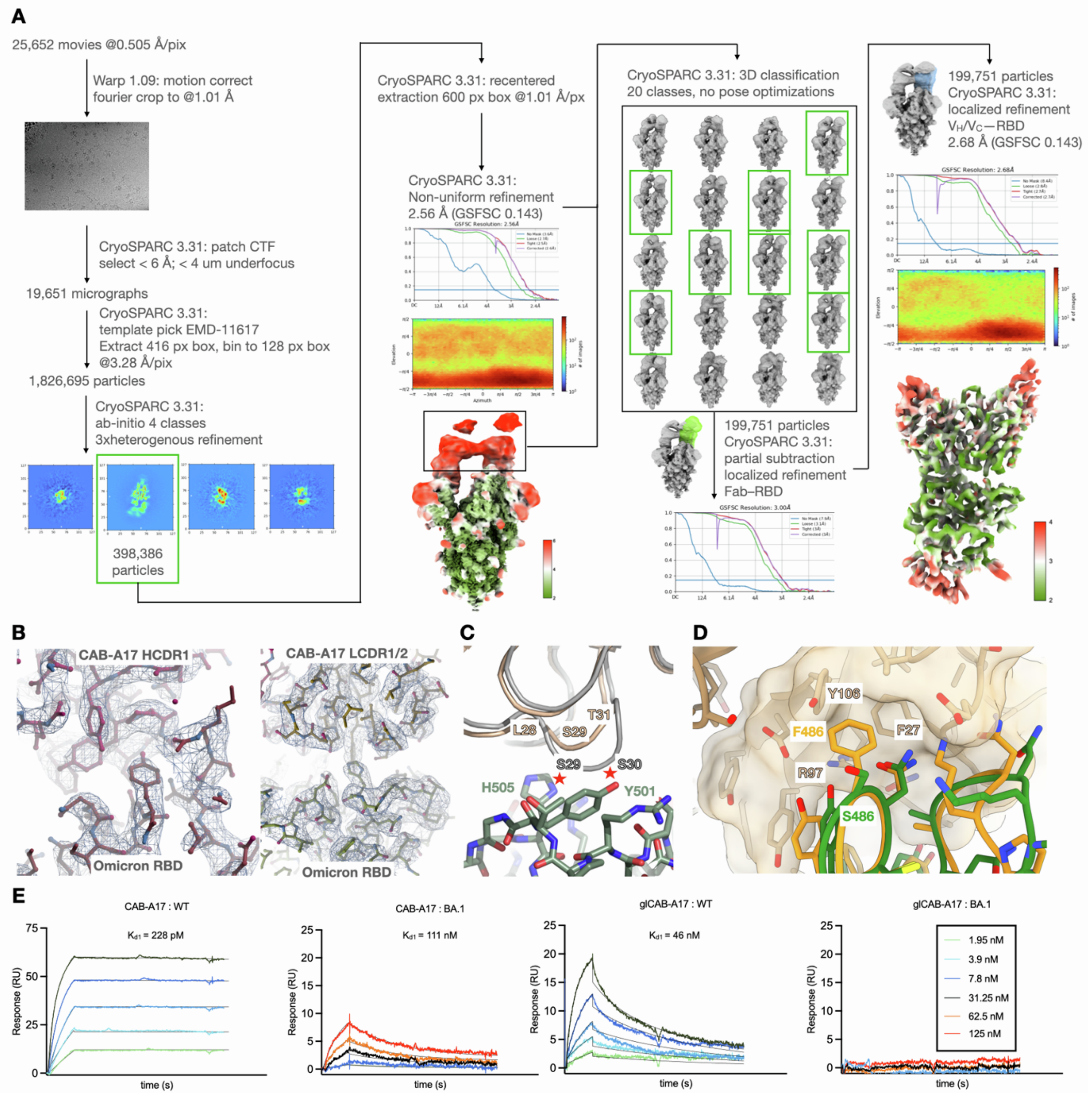

Figure S3. Cryo-EM processing workflow, light-chain shift and effects of F486S mutation. Related to Figures 2 and 3. **(A)** Cryo-EM processing workflow for the CAB-A17 Omicron spike complex. **(B)** Representative experimental map details. **(C)** The effect of the shortening of the CAB-A17 LCDR1 loop (CAB-A17 in light brown, C102 bound in grey). Potential sterical clashes denoted with a red star. **(D)** Difference in CAB-A17 interaction possibilities between BA.2.75 (orange-yellow;PDB:7YQY) and XBB (green; PDB:8IOU) due to an F486S mutation in XBB1. **(E)** Affinity of mature and germline-reverted CAB-A17 as measured using surface plasmon resonance (Biacore). Shown is the affinity of CAB-A17 IgG as well as its germline-reverted version (glCAB-A17) for immobilized RBD of the ancestral SARS-CoV-2 founder variant (WT) and Omicron BA.1 variant RBD (BA.1). Fitted (grey) is a bivalent binding model. K_{d1}, first dissociation rate constant. glCAB-A17 : BA.1 showed no affinity.

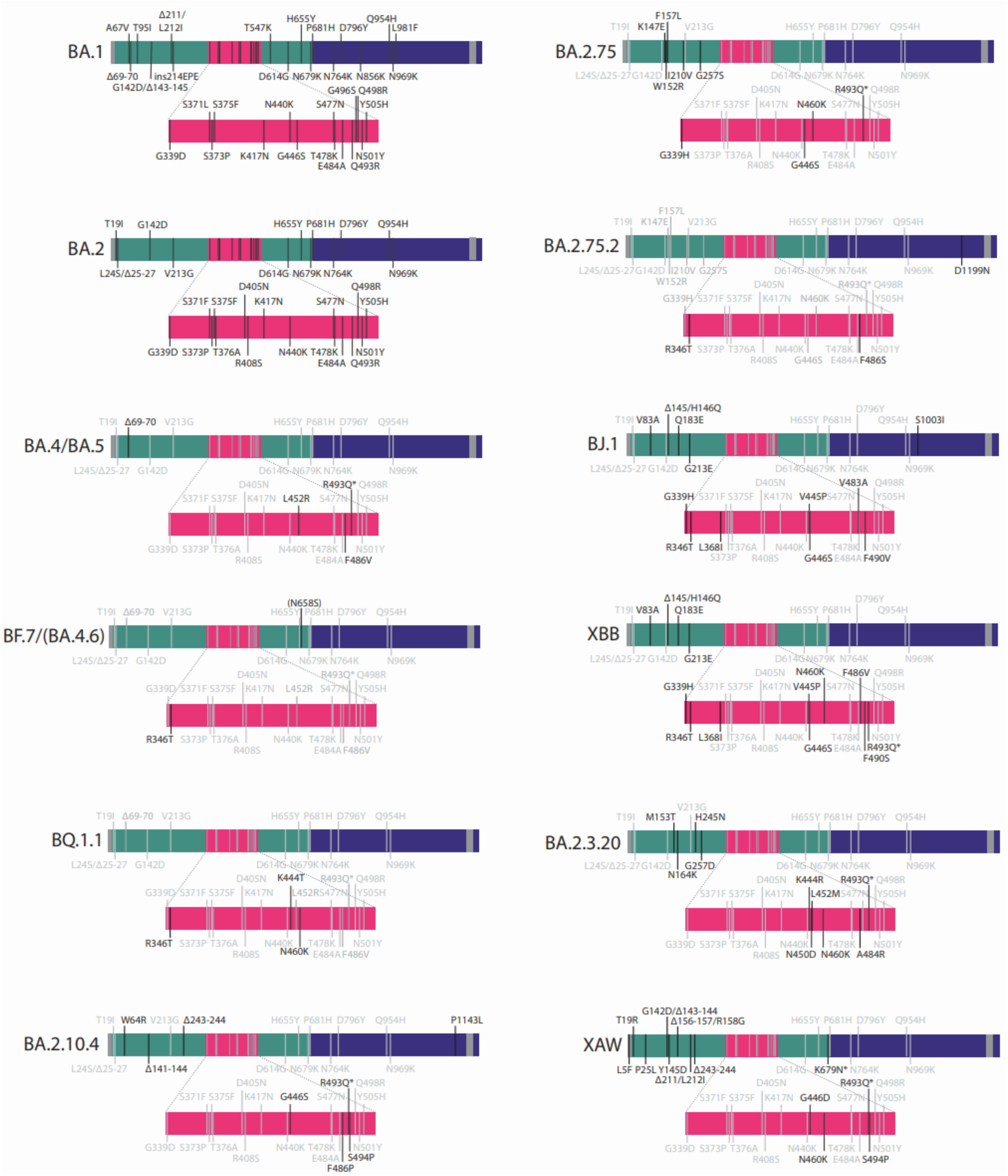

Figure S4. Schematic of the mutations in spike for the omicron sublineages forming the heterologous spike panel. Related to Figure 4.

Table S1. Cryo-EM data collection, refinement, and validation statistics. Related to Figure 2.

**Data collection**	Omicron spike + Fab CAB-A17 EMDB-16397 PDB 8C2R	Omicron + Fab CAB-A17 EMDB-42970 PDB 8V4F
Microscope	Krios G3i	-
Detector	K3 BioQuantum	-
Voltage (kV)	300	-
Electron exposure (e^-/Å^2)	54.6	-
Defocus range (μm)	–0.3 to –2.0	-
Frames	60	-
Magnification (nominal)	165,000	-
Final map pixel size (Å/px)	1.01	-
Symmetry imposed	C1	C1
No. of movies	25,652	-
No. of final particles images	398,386	199,751
Map resolution at 0.143 FSC threshold (Å)	2.56	2.68

Model refinement

Model composition

Non-hydrogen atoms	30,930	4,797
Protein residues	3,873	619
Non-protein residues	52 NAG	-

B-factors

Protein (Å^2)	80.7	84.9
Ligands (Å^2)	104	-

R.m.s. deviations

Bond lengths (Å)	0.007	0.002
Bond angles (°)	0.678	0.557

Validation[a]

MolProbity score	1.47	1.29
Clashscore	8.79	4.99
Poor rotamers (%)	0.00	0.19

Ramachandran plot

Favored (%)	98.42	97.87
Allowed (%)	1.58	2.13
Outliers (%)	0.00	0.00

[a] All validations statistics according to MolProbity[52]

Table S2. Polar-interaction table CAB-A17 – Omicron (Omicron vs Wu-Hu-1 mutations in red). Related to Figure 2.

CAB-A17 HC	Atom	Distance (Å)	Atom	Omicron RBD
E26	O	3.0	ND2	N487
E26	OE2	3.8 (salt bridge)	NZ	K478
I28	N	3.5	O	A475
A31	O	3.1	OH	Y473
N32	ND2	3.1	O	A475
Y33	OH	2.6	O	L455
G54	N	2.6	OH	Y421
G54	O	3.4	ND2	N460
S56	OG	2.7	OD2	D420
S56	OG	3.8	OD1	N460
R97	NH1	3.0	OD1	N487
R97	NH2	2.9	OH	Y489

CAB-A17 LC	Atom	Distance (Å)	Atom	Omicron RBD
G92	O	3.1	NE2	H505

Table S3. Bad clashes according to Molprobity[52] in the modeled C102 binding to Omicron (Omicron vs Wu-Hu-1 mutations in red). Related to Figure 2.

C102 HC	Atom	Atom	Omicron RBD
N32	OD1	HB1	A475
G54	N	OH	Y421
G54	CA	OH	Y421
G54	H	HH	Y421
R94	NH2	OH	Y489
Y99	OH	OH	Y453
Y99	CE2	NH1	R493
Y99	HE2	NH1	R493
G54	N	OH	Y421
G54	O	ND2	N460
Y102	CZ	CE1	F486
Y102	CE2	HE1	F486
**C102 LC**	**Atom**	**Atom**	**Omicron RBD**
S29	HG	ND1	H505
S29	OG	ND1	H505
S29	CB	ND1	H505
S29	CB	CG	H505
S29	HG	CG	H505
S29	HB2	CG	H505
S29	HB2	CE1	H505
S29	HB2	CD2	H505
S29	HG	CE1	H505
S29	CB	CE1	H505
S29	CB	CB	H505
S29	OG	CE1	H505
S29	HB3	CB	H505
S30	HA	OH	Y501
S30	HA	CZ	Y501
S30	CA	CZ	Y501
S30	OG	HB2	S496
G92	O	NE2	H505
G92	O	CE1	H505
