# Supplementary material for: Structural basis of broad SARS-CoV-2 cross-neutralization by affinity-matured public antibodies
Source: Cell Rep Med. 2024 May 17;5(6):101577. doi: 10.1016/j.xcrm.2024.101577 (PMC11228396; doi:10.1016/j.xcrm.2024.101577)
Supplement: Document S2. Article plus supplemental information [file mmc2.pdf]

Cell Reports Medicine

Report

Structural basis of broad SARS-CoV-2 cross-neutralization by affinity-matured public antibodies

Graphical abstract

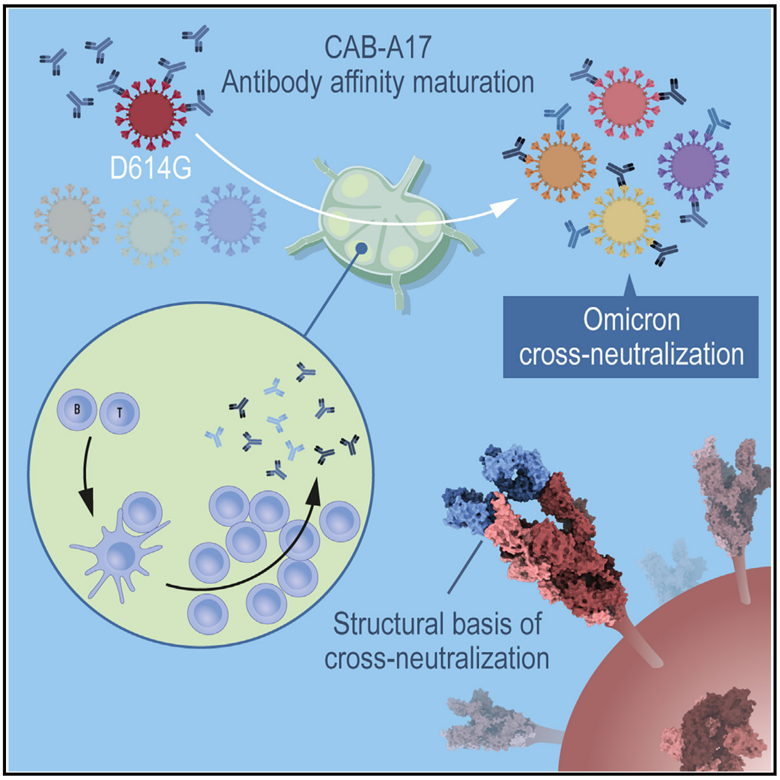

Highlights

- Some antibodies resulting from founder strain infection show a remarkable breadth
- Deep mutational scanning shows high resistance to viral escape
- Antibody affinity maturation was identified as key to developing breadth
- Cryo-EM reveals the basis for breadth developed through affinity maturation

Authors

Daniel J. Sheward, Pradeepa Pushparaj, Hrishikesh Das, ..., Ben Murrell, Gunilla B. Karlsson Hedestam, B. Martin Hällberg

Correspondence

benjamin.murrell@ki.se (B.M.),
gunilla.karlsson.hedestam@ki.se (G.B.K.H.),
martin.hallberg@ki.se (B.M.H.)

In brief

Sheward et al. find representative antibodies, raised following infection with the SARS-CoV-2 founder strain, that cross-neutralize Omicron variants. Combining deep mutational scanning, electron cryomicroscopy, and neutralization assays, they identify the structural basis for this cross-neutralization and reveal the role of somatic hypermutation in the broadening of the antibody response.

Sheward et al., 2024, Cell Reports Medicine *5*, 101577
June 18, 2024 © 2024 The Author(s). Published by Elsevier Inc.
https://doi.org/10.1016/j.xcrm.2024.101577

CellPress

Cell Reports Medicine

CellPress
OPEN ACCESS

Report

Structural basis of broad SARS-CoV-2 cross-neutralization by affinity-matured public antibodies

Daniel J. Sheward,[1,2,9] Pradeepa Pushparaj,[1,9] Hrishikesh Das,[3,9] Allison J. Greaney,[4] Changil Kim,[1] Sungyong Kim,[1] Leo Hanke,[1] Erik Hyllner,[1] Robert Dyrdak,[1] Jimin Lee,[4,8] Xaquin Castro Dopico,[1] Pia Dosenovic,[1] Thomas P. Peacock,[5] Gerald M. McInerney,[1] Jan Albert,[1] Martin Corcoran,[1] Jesse D. Bloom,[4,6] Ben Murrell,[1,10,*] Gunilla B. Karlsson Hedestam,[1,10,*] and B. Martin Hällberg[3,7,10,11,*]

[1]Department of Microbiology, Tumor and Cell Biology, Karolinska Institutet, Stockholm, Sweden
[2]Division of Medical Virology, Institute of Infectious Diseases and Molecular Medicine, University of Cape Town, Cape Town, South Africa
[3]Department of Cell and Molecular Biology, Karolinska Institutet, Stockholm, Sweden
[4]Basic Sciences Division and Computational Biology Program, Fred Hutchinson Cancer Research Center, Seattle, WA 98109, USA
[5]Department of Infectious Disease, Imperial College London, London, UK
[6]Howard Hughes Medical Institute, Chevy Chase, MD 20815, USA
[7]Centre for Structural Systems Biology (CSSB) and Karolinska Institutet VR-RÅC, Notkestraße 85, 22607 Hamburg, Germany
[8]Present address: Department of Biochemistry, University of Washington, Seattle, WA, USA
[9]These authors contributed equally
[10]Senior author
[11]Lead contact
*Correspondence: benjamin.murrell@ki.se (B.M.), gunilla.karlsson.hedestam@ki.se (G.B.K.H.), martin.hallberg@ki.se (B.M.H.)
https://doi.org/10.1016/j.xcrm.2024.101577

SUMMARY

Descendants of the severe acute respiratory syndrome coronavirus 2 (SARS-CoV-2) Omicron variant now account for almost all SARS-CoV-2 infections. The Omicron variant and its sublineages have spike glycoproteins that are highly diverged from the pandemic founder and first-generation vaccine strain, resulting in significant evasion from monoclonal antibody therapeutics and vaccines. Understanding how commonly elicited antibodies can broaden to cross-neutralize escape variants is crucial. We isolate IGHV3-53, using "public" monoclonal antibodies (mAbs) from an individual 7 months post infection with the ancestral virus and identify antibodies that exhibit potent and broad cross-neutralization, extending to the BA.1, BA.2, and BA.4/BA.5 sublineages of Omicron. Deep mutational scanning reveals these mAbs' high resistance to viral escape. Structural analysis via cryoelectron microscopy of a representative broadly neutralizing antibody, CAB-A17, in complex with the Omicron BA.1 spike highlights the structural underpinnings of this broad neutralization. By reintroducing somatic hypermutations into a germline-reverted CAB-A17, we delineate the role of affinity maturation in the development of cross-neutralization by a public class of antibodies.

INTRODUCTION

The Omicron variant,[1] comprising several highly diverged lineages, harbors numerous mutations in the viral spike relative to the pandemic founder, including many in the receptor-binding domain (RBD). As a result of this antigenic drift, Omicron lineages have escaped most monoclonal antibodies (mAbs) isolated to date[2–7] and display markedly reduced sensitivity to neutralization by serum from convalescent and vaccinated individuals.[6–14] However, a third dose with vaccines based on the founder spike significantly improved Omicron cross-neutralization,[15] with the expansion of affinity-matured cross-reactive memory B cells in the repertoire.[16] Understanding how antibodies elicited by the ancestral variant can bridge the gap to cross-neutralize Omicron is crucial in a pandemic now dominated by Omicron sublineages.

Several studies have characterized the B cell responses to severe acute respiratory syndrome coronavirus 2 (SARS-CoV-2) in the context of infection and vaccination and revealed that the immunoglobulin heavy chain variable 3-53 gene (IGHV3-53) was the most frequently used by neutralizing SARS-CoV-2 antibodies.[17,18] These antibodies display convergent features and typically fall within one of the two most common antibody "classes," categorized according to their targeted epitopes and characteristics.[19] "Class 1" antibodies are highly prevalent, and a majority use IGHV3-53 to bind to an epitope that overlaps the receptor-binding motif (RBM) that is only accessible in the RBD-up conformation. They typically possess short heavy-chain complementarity-determining regions (HCDR3s) that make only minor contributions to the antibody's interactions, and germline-encoded residues in IGHV3-53 dominate their interaction, enabling potent neutralization of the founder spike with minimal affinity maturation.[17] However, antibodies of this class frequently failed to cross-neutralize variants carrying mutations in the RBM.[20,21]

© 2024 The Author(s). Published by Elsevier Inc.
This is an open access article under the CC BY license (http://creativecommons.org/licenses/by/4.0/).

Here, we describe a panel of IGHV3-53-using mAbs isolated from a convalescent individual approximately 7 months after infection. We show that several of these were broadly cross-neutralizing, capable of neutralizing variants of concern (VoCs), including Omicron sublineages, with exceptional potency. By generating germline-reverted versions of one such antibody, CAB-A17, and introducing single or combinations of somatic hypermutations (SHMs) found in the mature antibody, we demonstrate that, while the initial antibody was largely strain specific, SHM at key positions enabled cross-neutralization of Omicron. Importantly, the introduction of only four SHMs enabled the cross-neutralization of Omicron with a potency equivalent to that of the germline antibody against D614G. Furthermore, we determined the high-resolution structure of trimeric Omicron spike in complex with CAB-A17 to define the structural basis for cross-neutralization of Omicron. This mechanistic understanding of Omicron cross-neutralization by public antibody lineages elicited by the ancestral SARS-CoV-2 variant may be crucial for developing improved vaccines capable of broad protection.

RESULTS

Identification of broadly cross-neutralizing IGHV3-53 antibodies

We expressed SARS-CoV-2 spike-specific mAbs isolated from memory B cells from a convalescent individual approximately 7 months following a primary infection and before vaccination.[22] The infection occurred in early 2020, during the first wave of the pandemic, likely with a Wu-Hu-1 or D614G-like virus.

Using a pseudovirus neutralization assay, we investigated the activity of the mAbs against ancestral B.1 (D614G) and a panel of VoCs, extending to Omicron BA.1 and BA.2. Several mAbs that used IGHV3-53 and had HCDR3 (Figure 1B) were similar to a class of previously described SARS-CoV-2-neutralizing antibodies.[17,23] However, they had higher levels of SHM than most previously described antibodies of this class. While several of the affinity-matured IGHV3-53-using mAbs showed a significant reduction in potency against BA.1 and BA.2, three mAbs from two clonally distinct lineages retained exceptional potency against these Omicron variants, with half-maximal inhibitory concentrations (IC_{50}) < 20 ng/mL but no mAb neutralized SARS-CoV (Figure 1C).

The antibody clonal lineages CAB-A (including mAbs CAB-A17 and CAB-A18) and CAB-B (with CAB-B37) displayed potent cross-neutralization of all SARS-CoV-2 VoCs tested, including the BA.1, BA.2 lineages of Omicron as well as other variants with varying degrees of resistance to antibody neutralization, including B.1.617.2 (Delta), B.1.351 (Beta), P.1 (Gamma), B.1.621 (Mu), and C.1.2 (Figure 1C). CAB-A49, a member of the broad CAB-A lineage but with fewer coding changes introduced by SHM than CAB-A17 and CAB-A18, also cross-neutralized all VoCs, albeit with reduced potency against several variants and with 7–14 times lower potency against BA.1 and BA.2 (Figure 1C).

From other lineages, CAB-C19 retained neutralization potency against all pre-Omicron VoCs but had reduced potency against Omicron BA.1 (7-fold lower) and BA.2 (47-fold lower). Similarly, CAB-D16 cross-neutralized all other VoCs but lacked any detectable neutralizing activity against Omicron. From lineage E, both CAB-E24 and CAB-E30 cross-neutralized Alpha, Delta, C.1.2, and Mu but had reduced potency against Beta and Gamma and showed only weak cross-neutralization of BA.1 and BA.2 (Figure 1C). Like Omicron, the RBDs of Beta and Gamma are mutated at amino acid position 417, which is a critical contact residue for antibodies of this class.[27–29]

Deep mutational scanning revealed that CAB-A17 is highly resistant to mutational escape

With the continued transmission and evolution of SARS-CoV-2, the emergence of variants with substantial resistance to antibody-mediated neutralization undermines the protection afforded by vaccine-induced responses and the effect of mAb therapeutics. To characterize the mode of recognition and escape landscape for the broadly neutralizing antibodies isolated here, we applied deep mutational scanning (DMS). Using a yeast-displayed mutant RBD library, we comprehensively mapped RBD mutations (in the eliciting Wu-Hu-1 background) that could endow resistance to neutralization by CAB-A17-like antibodies.[30] We similarly mapped the escape landscape of CAB-A49, a less broad member of the same lineage, and CAB-C19, an antibody of the same class from a different lineage that displayed only moderately potent cross-neutralization of Omicron.

Consistent with the relative neutralization breadth of these antibodies, multiple potential escape mutations were identified for CAB-A49 and CAB-C19. These were mainly localized to spike residues 417, 420, 456, and 475, but CAB-A17 was highly resistant to escape (Figures 1D and 1E). Only two mutations were identified as significant contributors to the escape fraction for CAB-A17: A475N, which introduces an N-linked glycosylation sequon, and F456K.

We sought to confirm these results in pseudovirus neutralization assays, introducing these mutations by site-directed mutagenesis. A spike carrying the A475N mutation did not generate pseudoviruses capable of efficient infection, potentially due to the introduction of an N-linked glycan adjacent to the RBM and, consistent with this mutation, only observed three times in deposited sequences (and all three flagged with sequence quality concerns). The introduction of F456K yielded a functional pseudovirus, which CAB-A17 neutralized with ~3-fold reduced potency (Figure 1E). Thus, the single mutation identified to afford the highest level of binding escape against CAB-A17 in the DMS analysis only led to a modest reduction in neutralizing activity, demonstrating that CAB-A17 is highly resistant to mutational escape in the context of the eliciting antigen. The introduction of F456K only moderately reduced neutralization by CAB-C19 but afforded substantial resistance to neutralization by CAB-A49 (Figure 1E), in robust concordance with the prevalence of this mutation in the escape fraction for each mAb in the DMS analysis.

Cryo-EM of CAB-A17 in complex with the Omicron spike

To define the structural context underpinning the cross-neutralization of Omicron, we resolved the structure of a CAB-A17 Fab in a complex with the Omicron (BA.1) spike. The cryo-EM

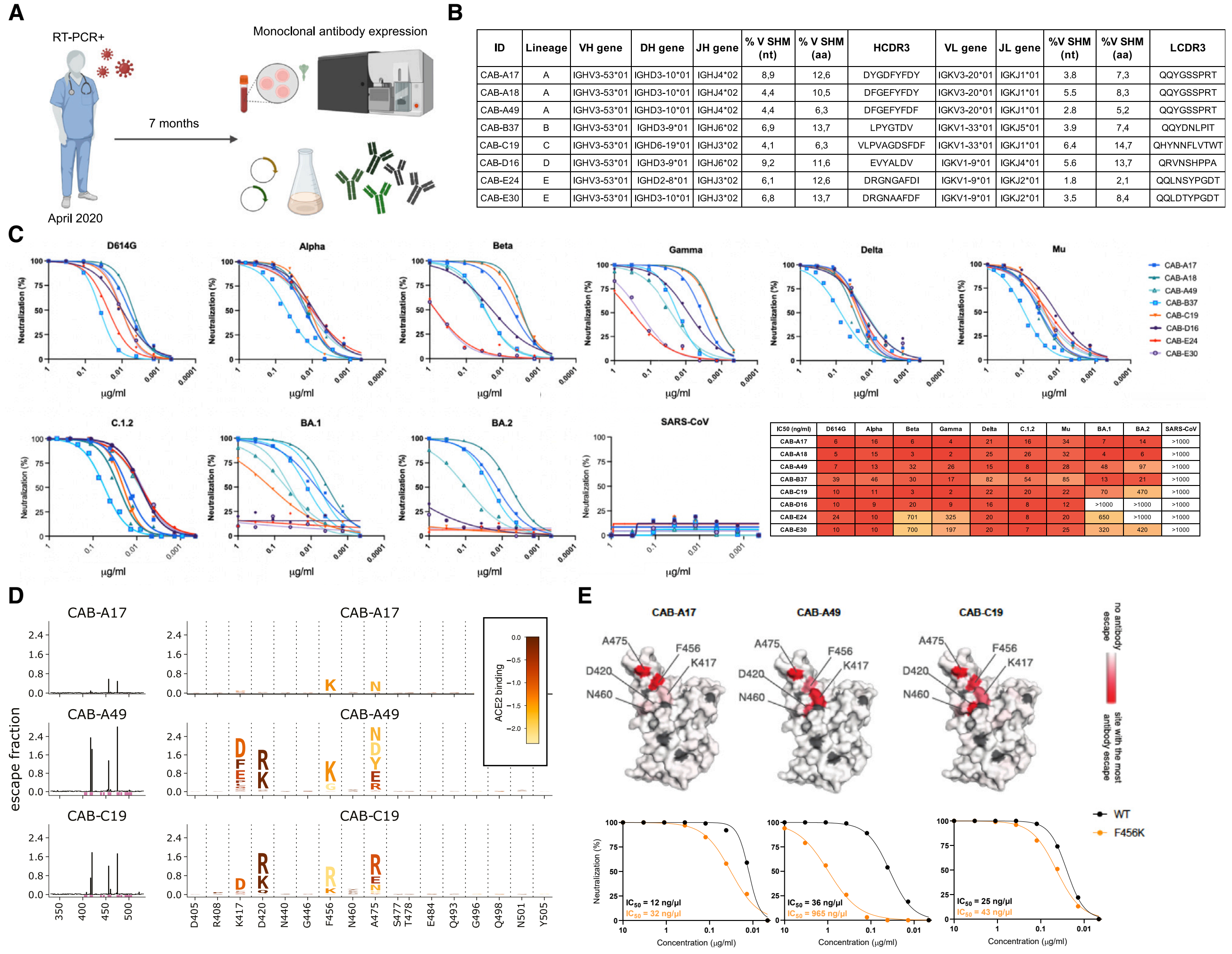

ID	Lineage	VH gene	DH gene	JH gene	% V SHM (nt)	% V SHM (aa)	HCDR3	VL gene	JL gene	%V SHM (nt)	%V SHM (aa)	LCDR3
CAB-A17	A	IGHV3-53*01	IGHD3-10*01	IGHJ4*02	8,9	12,6	DYGDFYFDY	IGKV3-20*01	IGKJ1*01	3.8	7,3	QQYGSSPRT
CAB-A18	A	IGHV3-53*01	IGHD3-10*01	IGHJ4*02	4,4	10,5	DFGEFYFDY	IGKV3-20*01	IGKJ1*01	5.5	8,3	QQYGSSPRT
CAB-A49	A	IGHV3-53*01	IGHD3-10*01	IGHJ4*02	4,4	6,3	DFGEFYFDF	IGKV3-20*01	IGKJ1*01	2.8	5,2	QQYGSSPRT
CAB-B37	B	IGHV3-53*01	IGHD3-9*01	IGHJ6*02	6,9	13,7	LPYGTDV	IGKV1-33*01	IGKJ5*01	3.9	7,4	QQYDNLPIT
CAB-C19	C	IGHV3-53*01	IGHD6-19*01	IGHJ3*02	4,1	6,3	VLPVAGDSFDF	IGKV1-33*01	IGKJ1*01	6.4	14,7	QHYNNFLVTWT
CAB-D16	D	IGHV3-53*01	IGHD3-9*01	IGHJ6*02	9,2	11,6	EVYALDV	IGKV1-9*01	IGKJ4*01	5.6	13,7	QRVNSHPPA
CAB-E24	E	IGHV3-53*01	IGHD2-8*01	IGHJ3*02	6,1	12,6	DRGNGAFDI	IGKV1-9*01	IGKJ2*01	1.8	2,1	QQLNSYPGDT
CAB-E30	E	IGHV3-53*01	IGHD3-10*01	IGHJ3*02	6,8	13,7	DRGNAAFDF	IGKV1-9*01	IGKJ2*01	3.5	8,4	QQLDTYPGDT

IC50 (ng/ml)	D614G	Alpha	Beta	Gamma	Delta	C.1.2	Mu	BA.1	BA.2	SARS-CoV
CAB-A17	6	16	6	4	21	16	34	7	14	>1000
CAB-A18	5	15	3	2	25	26	32	4	6	>1000
CAB-A49	7	13	32	26	15	8	28	48	97	>1000
CAB-B37	39	46	30	17	82	54	85	13	21	>1000
CAB-C19	10	11	3	2	22	20	22	70	470	>1000
CAB-D16	10	9	20	9	16	8	12	>1000	>1000	>1000
CAB-E24	24	10	701	325	20	8	20	650	>1000	>1000
CAB-E30	10	10	700	197	20	7	25	320	420	>1000

Figure 1. Identification of broadly cross-neutralizing SARS-CoV-2 antibodies and DMS

(A) Schematic overview of the isolation and expression of mAbs.

(B) Genetic features of the SARS-CoV-2 mAbs characterized here. Sequence alignments toward inferred gls are shown in Figure S1.

(C) Cross-neutralization of variants D614G (B.1), Alpha (B.1.1.7), Beta (B.1.351), Gamma (P.1), Delta (B.1.617.2), C.1.2, Mu (B.1.621), Omicron (BA.1 and BA.2), and SARS-CoV by isolated mAbs.

(D) The sum of the antibody escape fraction for all mutations per site on antibody binding by DMS. The logo plots on the right show key sites, with the height of each letter corresponding to that mutation's escape fraction. Letters are colored according to that mutation's effect on ACE2 binding, as measured by Starr et al.[24]

(E) The site-total antibody escape is mapped to the surface of the Wuhan-Hu-1 RBD (PDB: 6M0J), with white indicating no escape and red indicating the site with the most antibody escape. Sites where no mutations are tolerated are gray. Shown are neutralization curves of D614G (wild type [WT], black) and D614G + F456K (F456K, orange) pseudotyped viruses by mAbs CAB-A17, CAB-A49, and CAB-C19. All escape scores can be found on GitHub.[25] Interactive versions of logo plots and structural visualizations are from GitHub.[26] The complete mapping of mutations that escape antibody binding to the Wuhan-Hu-1 RBD is shown in Figure S2.

reconstruction, with an overall resolution of 2.6 Å, revealed a 2-up conformation of the spike, and each up-RBD was decorated with a CAB-A17 antibody fragment (Fab) bound to the ACE2 binding surface with a mode of recognition typical of "class 1" antibodies[23,29] (Figure 2A). The observed CAB-A17 Fab binding mode was compatible with the bivalent binding of full immunoglobulin G (IgG) to the Omicron spike.

To obtain a molecular understanding of the CAB-A17-Omicron RBD interaction, we used localized reconstruction techniques on a subset of the particles and thereby obtained a 2.7-Å resolution reconstruction of the paratope-epitope interface (Table S1; Figures S3A–S3B). All CDRs of the light and heavy chains of CAB-A17 interacted with the Omicron RBD (Figure 2B) through surface complementarity and specific hydrogen bonds (Table S2). Remarkably, seven of the interaction-interface residues on the Omicron spike are mutated relative to the founder variant (Figure 2C).

To better resolve the interactions enabling cross-neutralization of Omicron by CAB-A17, we compared the interactions with those of the mAb C102. C102 and CAB-A17 are both class 1 antibodies[23] and have similar binding modes[19] (PDB: 7K8M), with a C_α root-mean-square deviation (RMSD) of 0.62 $Å^2$ when

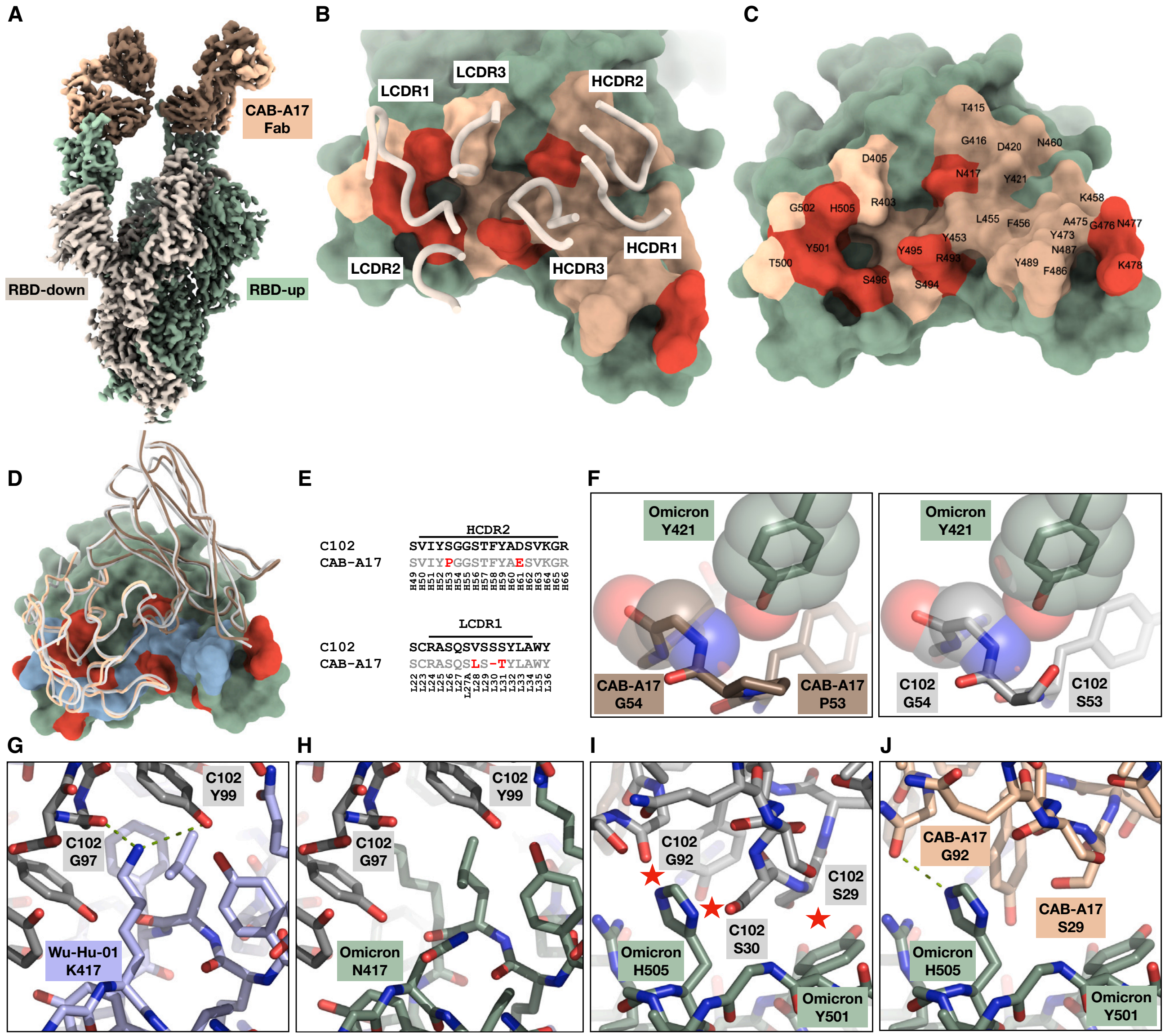

Figure 2. Cryo-EM structure of the CAB-A17 Omicron-spike complex and comparison between CAB-A17 and C102

(A) Cryo-EM reconstruction of CAB-A17 Fab bound to the Omicron BA.1 spike determined to an overall resolution of 2.6 Å. For illustration and clarity purposes, the map is a composite of the local reconstruction (2.7 Å) and the overall reconstruction. Protomers in the RBD-up conformation are colored green and light green. The RBD-down conformation protomer is colored in warm gray. The CAB-A17 Fab light and heavy chains are colored light and dark brown, respectively.

(B) The epitope of CAB-A17 mapped on the Omicron RBD interface. Light and heavy CDR loops are labeled. Epitope residues contacting the light and heavy chains are shown in light brown and dark brown, respectively. Residues that are mutated in the Omicron variant are labeled red-orange.

(C) Epitope residues labeled. The same coloring as in (B) is used.

(D) ACE2-Omicron RBD interface, with ACE2-contacting residues labeled in light blue and residues mutated in Omicron labeled red-orange, overlayed with a superimposition of CAB-A17 and C102. C102 is colored light gray. The CAB-A17 Fab light and heavy chains are colored light and dark brown, respectively.

(E) Alignment showing differences between C102 and CAB-A17 in HCDR2 and LCDR1.

(F) Modeling of the interaction between Y421 and P53/S53/G54 in HCDR2. Relevant atoms are shown in both stick and transparent VDW sphere representation.

(G and H) K417 in the founder variant (Wu-Hu-1) forms two strong hydrogen bonds to the C102 G97 and Y99 (G, left), while these are lost in Omicron through the K417N mutation (H).

(I and J) Interactions in LCDR1. In C102, S30 and G92 clash with Omicron H505, and S29 clashes with Omicron Y501 (I), while CAB-A17 G92 forms a hydrogen bond with Omicron H505 (J).

aligned to their respective RBDs (Figure 2D). C102 also uses the same light-chain gene (IGKV3-20) and has 91% amino acid identity to the CAB-A17 heavy chain. However, while C102 potently neutralizes the SARS-CoV-2 founder and Delta (IC_{50} = 52 ng/mL and 17 ng/mL, respectively), it fails to neutralize Beta and Omicron (IC_{50} > 10 μg/mL[31]).

Modeling C102 with the Omicron RBD reveals several potential clashes (Table S3). While we estimate that most of these

CellPress
OPEN ACCESS

clashes would require only slight shifts of side-chain conformations, one potential clash was identified—in HCDR2, specifically around residue 53 (S53 in C102 vs. P53 in CAB-A17; Figure 2E)—that would require main-chain shifts. The K417N mutation present in Omicron also removes two strong hydrogen bonds from the interaction between C102 HCDR3 (Y99 and G97) and the founder variant RBD (Figures 2G and 2H).

For the C102 light chain, there are several potential clashes when binding Omicron in the region close to the mutated Q498R, N501Y, and Y505H in the LC interaction area (Figure 2I; Table S3). In contrast, CAB-A17 LCDR1 SHMs, including a 1-amino acid (aa) deletion, changed the germline VSSS motif present in C102 to an LS-T (Figure 2E). This led to a slight shortening of the backbone in a critical region close to the mutated Q498R, N501Y, and Y505H in the LC interaction area (Figure S3C), enabling CAB-A17 to retain a favorable RBD binding mode (Figure 2J; Table S2). However, even without the LCDR1 deletion, binding in this region may still occur, but with a change in the Ab-RBD approach angle or other rearrangements of the paratope.

Affinity maturation enabled cross-neutralization

Many IGHV3-53-using mAbs isolated to date are capable of potent ancestral SARS-CoV-2 neutralization with only low levels of SHM, as important recognition motifs are germline-encoded by the IGHV gene. However, these antibodies are typically strain specific and fail to cross-neutralize Omicron. The CAB-A17 IGHV region contained a higher level of SHM compared to other lineage members, with several coding changes in amino acids located in or near the HCDRs (Figure S1). This, together with the comparisons to the C102 structure, suggested that the breadth of CAB-A17 was attributable to its affinity maturation, particularly in and around CDRH1 and CDRH2.

To precisely resolve the role of SHM in the cross-neutralization of Omicron, we reverted the heavy and light chains of the mature CAB-A17 to their germline forms while keeping the HCDR3s intact (germline CAB-A17 [glCAB-A17]). While glCAB-A17 could neutralize D614G, albeit 55-fold less potently than the mature antibody, it lacked neutralizing activity against Beta and Omicron (BA.1) (Figures 3A and 3B), confirming the essential role of affinity maturation in the acquisition of breadth. Reversion of only the LC SHMs to the gl configuration (mHC-glLC) reduced the potency against D614G by approximately 3-fold and against Beta and BA.1 by about 10-fold, but all variants were still potently neutralized. In contrast, the reversion of only the HC SHMs to the gl configuration (glHC-mLC) abolished the neutralization of Beta and BA.1 as well as of pseudoviruses just carrying K417N (Figure 3B). Taken together, these data highlight that affinity maturation led to the broadening of CAB-A17 and that mutations in the heavy chain played a dominant role in enabling this breadth. Indeed, surface plasmon resonance measurements show that, while the gl-reverted antibody had an undetectable affinity for the Omicron spike, the mature antibody had an affinity approaching that of the gl-reverted antibody against the ancestral variant (Figure S3E).

Structural basis for enhanced neutralization through affinity maturation

To define the roles of individual heavy-chain SHMs, we introduced select SHMs, individually or in combination, into glCAB-A17. These included SHMs present in all three antibodies of the lineage (G26E, T28I, Y58F) or present in the two broadest members, CAB-A17 and CAB-A18, but not in CAB-A49 (S31 R/A and S53P) (Figure S1). Each of these introduced mutations enhanced the neutralization of D614G, but none enabled detectable cross-neutralization of BA.1 on their own (Figure 3B). However, introducing four SHMs in the HC together (G26E, T28I, S53P, and Y58F) enabled cross-neutralization of BA.1 equivalent to the gl antibody against D614G and enabled potency against D614G approaching that of the mature antibody (Figure 3B).

Comparison of the cryo-EM structure of CAB-A17 to C102 identified a potential role of the shortening of the light chain. Thus, to determine whether SHMs in the light chain synergized with those in the heavy chain, we expressed the heavy-chain variants paired with the mature CAB-A17 light chain. While SHMs in the light chain afforded substantial improvements in the neutralization of K417N and Beta, they did not further improve the neutralization of BA.1 over and above G26E, T28I, S53P, and Y58F (Figure 3B).

To resolve the influence of these SHMs on the structure of CAB-A17 and its interactions with the RBD, we additionally leveraged representative gl IGHV3-53 structures (PDB: 5ILH and 5ILE)[32] that are identical in sequence to glCAB-A17 outside HCDR3.

In the HCDR1 in CAB-A17, G26E limits the conformational variability of the loop and promotes an HCDR1-stretch main-chain position more distant to the RBD than in the gl and unbound C102. Furthermore, G26E promotes a rotamer switch of F27 from the p90 rotamer in the gl and unbound C102 to the m-85 rotamer found in CAB-A17 and C102 when bound to the RBD (Figure 3D). In addition, T28I and S31A in CAB-A17 remove the possibility of a T28-S31 hydrogen bond that is present in the gl antibody. This hydrogen bond stabilizes the 28–31 turn in a position where residue 31 clashes with the sterically locked RBD Y473 (Figure 3D). Furthermore, in the HCDR2 region, the S53P SHM removes backbone conformational variability and prevents the HC-53 backbone carbonyl from the peptide-plane flip observed in gl, where it would clash with RBD Y421 (Figure 3E). Furthermore, the HCDR2 Y58F hinders a clash with RBD T415 (Figure 3E). By analyzing entries utilizing V3-53 and V3-66 gls in the CoV-AbDab database,[33] we find that 9% of these antibodies have G26E, 25% have T28I, and 58% have Y58F. Despite requiring two nucleotide changes, 10% of sequences have S53P. 53P occurs 13 times with 58F and only four times with Y58. Hence, the S53P change occurs preferentially with Y58F, with Y58F more commonly observed versus S53P. This makes structural sense because the Y58F SHM enables a tighter RBD-HCDR2 interaction and is also present in C102 (Figure 3C).

For the LCDR1, the combination of an SHM-driven loop shortening, in combination with S31T in the loop turn that provides a hydrogen bond between T31 and the backbone carbonyl of S30, provides a significantly more rigid LCDR1 loop conformation

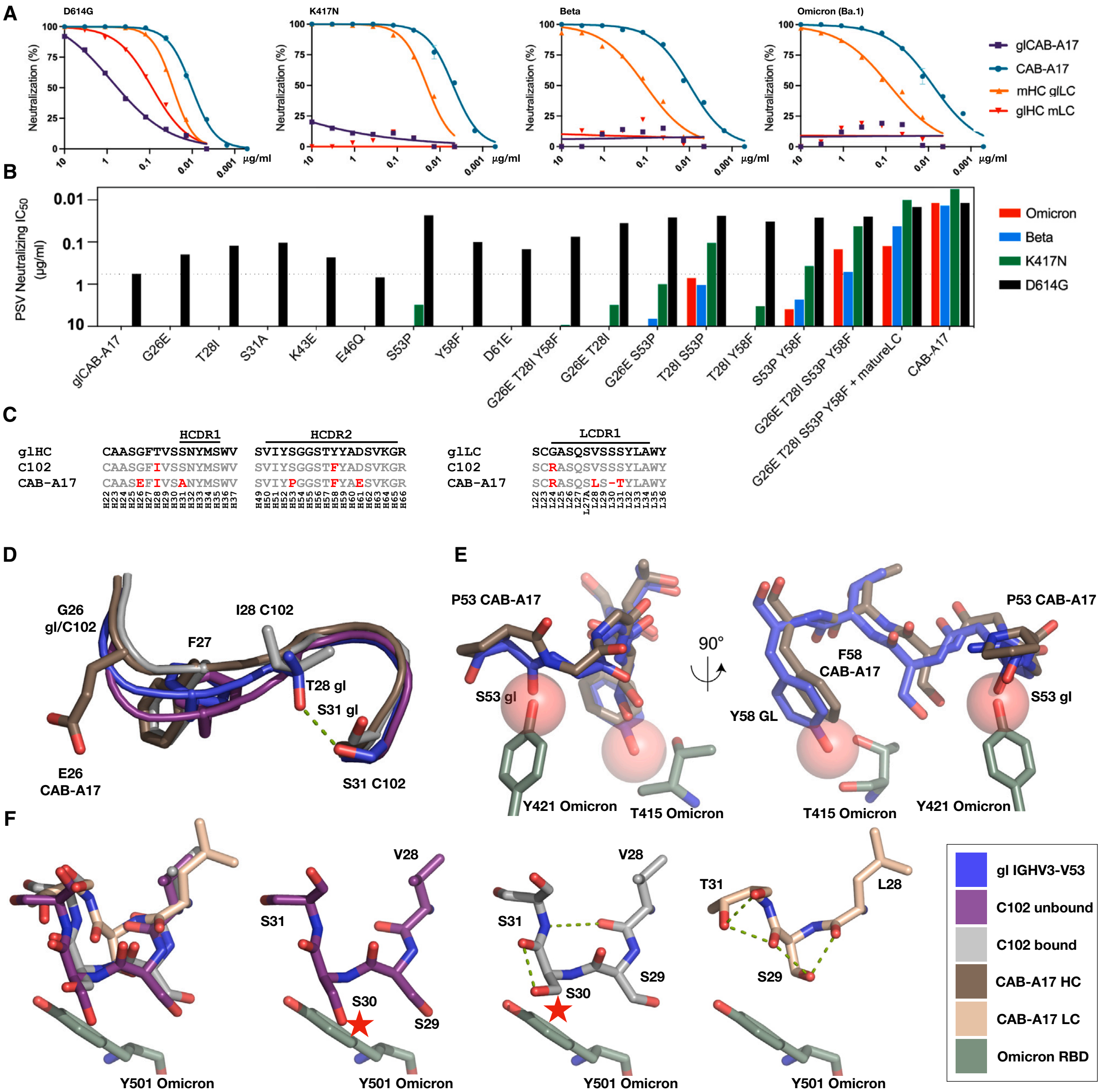

Figure 3. Affinity maturation enabled the cross-neutralization of Omicron and its structural basis

(A) Pseudovirus neutralization by versions of CAB-A17, with SHMs reverted in either the heavy (glHC mLC, red) or light chain (mHC glLC, orange), compared to the fully mature antibody (CAB-A17, teal) and the fully gl-reverted (apart from the CDR3) version (glCAB-A17, purple), demonstrating a dominant role of HC SHM.

(B) Summary of neutralizing IC_{50} values, demonstrating the effect of the introduction of identified SHMs into the glCAB-A17 HC on the neutralization of D614G (B.1) (black), Beta (blue), D614G + K417N (K417N, green), and Omicron (red).

(C) Alignment of gl-LC/HC, C102, and CAB-A17, showing the key LC and HC SHMs.

(D) Structural alignment of HCDR1 of a representative gl IGHV3-V53 (5i1h), unbound C102 (7k8n), RBD-bound C102 (7k8m), and CAB-A17 (8c0y).

(E) Structural alignment of HCDR2 of a representative gl IGHV3-V53 (5i1e) and CAB-A17 (8c0y).

(F) Structural alignment of LCDR1 of unbound C102 (7k8n), RBD-bound C102 (7k8m), and CAB-A17 (8c0y). Coloring throughout is according to the color key (bottom right). Red stars denote steric clashes.

CellPress
OPEN ACCESS

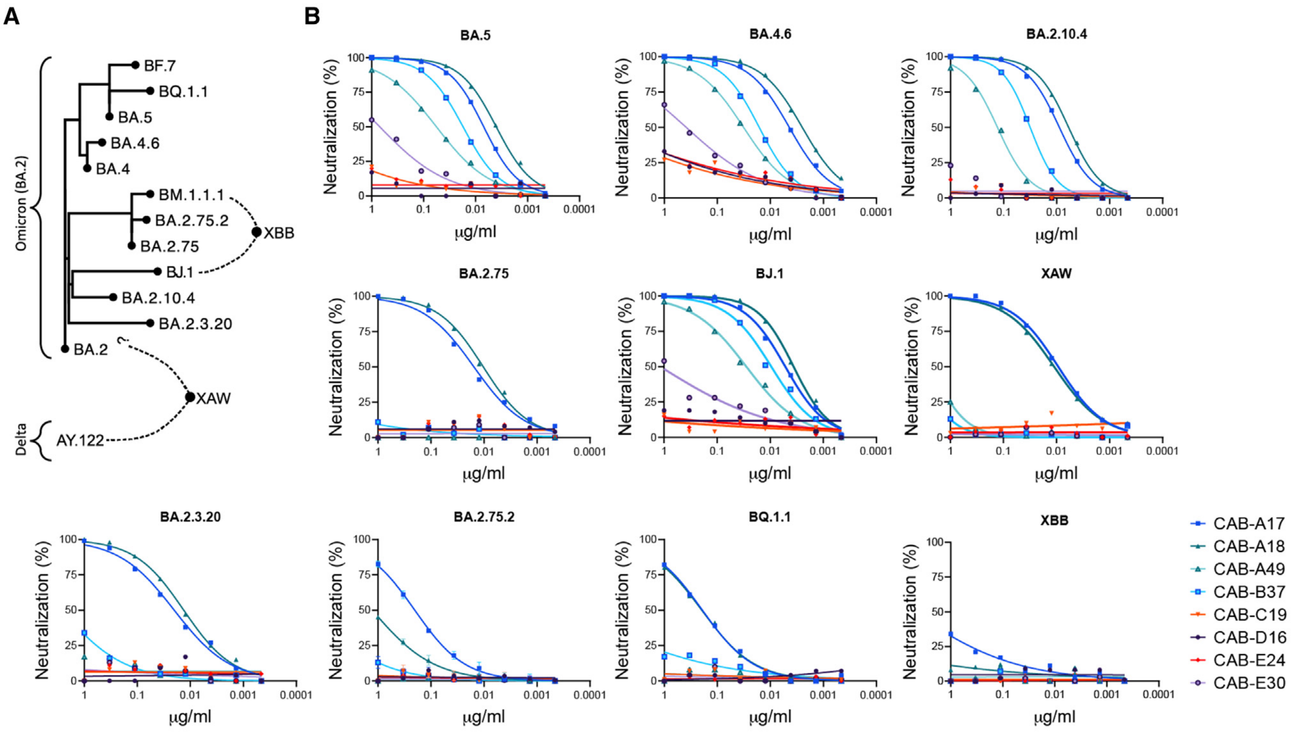

IC50 (ng/ml)	BA.5 (BA.4)	BA.4.6	BA.2.10.4	BA.2.75	BJ.1	XAW	BA.2.3.20	BA.2.75.2	BQ.1.1	XBB
CAB-A17	7	4	9	15	5	9	20	202	190	>1000
CAB-A18	4	2	6	10	4	11	13	1282	196	>1000
CAB-A49	57	32	131	>1000	28	>1000	>1000	>1000	>1000	>1000
CAB-B37	17	16	32	>1000	10	>1000	>1000	>1000	>1000	>1000
CAB-C19	>1000	>1000	>1000	>1000	>1000	>1000	>1000	>1000	>1000	>1000
CAB-D16	>1000	>1000	>1000	>1000	>1000	>1000	>1000	>1000	>1000	>1000
CAB-E24	>1000	>1000	>1000	>1000	>1000	>1000	>1000	>1000	>1000	>1000
CAB-E30	696	385	>1000	>1000	>1000	>1000	>1000	>1000	>1000	>1000

Figure 4. Broad cross-neutralization of emerging Omicron sublineages

(A) Phylogeny demonstrating the diversity in spike of emerging Omicron sublineages. Spike mutations in the panel of variants evaluated here are summarized in Figure S4.

(B) Cross-neutralization of variants BA.5, BA.4.6, BA.2.7.5, BA.2.10.4, BJ.1, XAW, BA.2.75.2, BA.2.3.20, XBB, and BQ.1.1 by the isolated mAbs presented in this work.

than in C102/gl (Figure 3F). This may explain the lack of full restoration of neutralization activity even after reintroducing the four key HC SHMs into glCAB-A17 (Figure 3B).

Together, these data highlight the structural basis by which relatively few SHMs confer breadth to a commonly elicited class of IGHV3-53 antibodies extending to the highly divergent Omicron lineage.

Affinity-matured antibodies resulting from infection by the founder strain neutralize recent Omicron sublineages

To determine the extent of the breadth that affinity maturation provided, we evaluated the cross-neutralization of more recently emerged sublineages of Omicron (Figure 4A), representing over 2 years of evolution from the antigen that elicited the response.

CAB-A17, CAB-A18, and CAB-B37 all cross-neutralized BA.5 and BA.4.6 without a significant loss of potency (Figure 4B). The BA.2/Delta recombinant, XAW, was potently neutralized by the two broadest antibodies, CAB-A17 and CAB-A18, as were BJ.1 and BA.2.3.20. Two of the most resistant spike variants identified to date, BA.2.75.2 and BQ.1.1,[11,13] were still neutralized by CAB-A17, albeit only at an IC_{50} of ~200 ng/mL. XBB was highly resistant to neutralization by all mAbs, including CAB-A17, in line with its extremely low sensitivity to serum neutralization, likely attributable to F486S (Figure S3D).[11,13]

DISCUSSION

The emergence of the Omicron lineage was accompanied by extensive resistance to neutralizing antibodies,[2,3,7,8] where few mAbs elicited by primary vaccination or infection maintained potency against Omicron sublineages.[4,6,9–11,13] Here, we describe several potently neutralizing IGHV3-53-using antibodies targeting the RBM, capable of Omicron cross-neutralization, extending to BA.2 and even BA.4/BA.5 sublineages. In direct comparisons, CAB-A17 was 15-fold more potent against Omicron (BA.1) than S309 (sotrovimab)[7] and had potency comparable to the ultrapotent LY-CoV1404 (bebtelovimab).[34] CAB-A17 and the clonally related CAB-A18 use IGHV3-53 and IGHJ4, have a short (9-aa) HCDR3, and are paired with IGKV3-20, all typical of common "class 1"[19] antibodies (public clonotype 2).[18]

All currently known alleles of IGHV3-53 contain the gl-encoded motifs in HCDR1 and HCDR2 (NY and SGGS, respectively) that enable their interaction with the class 1 epitope in the SARS-CoV-2 RBM. The closely related IGHV3-66 gene similarly contains these motifs.[18,29] IGHV3-53 is highly overrepresented among potent SARS-CoV-2-neutralizing mAbs isolated early in the pandemic.[17,35–37] The precursor frequency of this class of neutralizing antibodies in the human repertoire was estimated to be around 1: 44,000,[38] suggesting that such antibodies are readily elicited in most people.

CAB-A17 and CAB-A18, described here, were more affinity matured (V gene SHM: 12.6% aa, 8.9% nucleotide, and 10.5% aa, 4.4% nucleotide, respectively) than the previously described prototypical members of this class, likely reflecting the relatively delayed isolation, 7 months following a confirmed SARS-CoV-2 infection. CAB-A49 from the same lineage but with fewer aa SHMs (6.3%) did not exhibit the same breadth, suggesting the importance of SHM in developing breadth. However, only four SHMs in the HC alone were required to neutralize Omicron with the same potency as the gl antibody against D614G, indicating that the mutational barrier to breadth is not prohibitive. This suggests that specific, but not extensive, SHM pathways are required for breadth by this antibody class.

We used DMS to better resolve the RBD escape landscape in the context of the eliciting antigen. We found that CAB-A17 was highly resistant to escape, where no single mutation introduced into the ancestral RBD afforded significant escape. Even the single mutation with the highest escape (F456K) afforded only modestly reduced sensitivity to neutralization for Wu-Hu-1. While escape from these and similar antibodies may be afforded by F456K, mutations at this site may reduce the affinity of the RBD for ACE2.[24] Furthermore, an F-to-K mutation requires three nucleotide changes, and, excluding low-coverage sequences, this mutation (F456K) has only been observed in two sequences deposited in GISAID29 to date (October 2023), and both are labeled as "under investigation," suggestive of low sequencing quality. As noted above, the DMS was performed in the background of the ancestral RBD, and therefore, additional mutations and epistatic interactions may alter the escape landscape in the context of the Omicron RBD. Indeed, mutation to a leucine at this position (F456L) recently emerged in several lineages and is increasing in frequency, which likely reflects escape from similar cross-neutralizing antibodies at the population level.

The cryo-EM structure of CAB-A17 in complex with the Omicron spike sheds light on the structural basis of its broad SARS-CoV-2 cross-neutralization. From modeling and analysis of available structures of gl IGHV3-53 and a representative IGHV3-53 Ab that neutralizes Wu-Hu-1 but not Omicron (C102), considerable conformational changes are necessary to enable binding to the Wu-Hu-1 RBD in general and the Omicron RBD in particular. Such conformational changes in the paratope incur a high entropic cost of binding. Based on the structural comparisons, the neutralization capacity gain during affinity maturation for the most important SHMs of CAB-A17 can be understood structurally within the framework of reduced entropy cost for binding through the prearrangement of an Ab paratope.[39] Specifically, the G26E, T28I, and S31A SHMs in HCDR1 preconfigure the loop in a conformation compatible with RBD binding. A similar but most likely not as effective preconfiguration of HCDR1 can be achieved with an F27I SHM (as in CAB-A49 here) that would not require a rotamer shift. In line with this, F27I SHM is critical for the potency of P2C-1F11 (Brii-196).[40] Likewise, S53P preconfigures HCDR2 for RBD binding by locking the loop in a conformation optimal for Y421 interaction. Interestingly, in all gl IGHV3-53 and IGHV3-66 alleles, the serine at position 53 is encoded by the codon AGC, which requires at least two nucleotide changes to encode a proline. This mutational barrier likely explains why the S53P mutation, which offers the largest improvement in neutralization potency against D614G of all single mutations tested, is not observed more frequently. The fact that S53P is almost always observed together with Y58F can be understood structurally, as Y58F enables a tight, high-affinity association between the HCDR2 and the RBD. Without Y58F, S53P is of limited utility for locking the HCDR2 in an RBD-binding-compatible conformational prearrangement.

Light-chain interactions also play critical roles in neutralizing the Beta and Gamma variants by this antibody class, where the LCDR1 of these antibodies make direct contact with the spike near residue 501. In line with this, the light chain from COVOX-222 could rescue the neutralization of Beta and Gamma by other IGHV3-53 antibodies.[20] Omicron BA.1 carries not only N501Y but also proximal mutations at 496, 498, and 505. CAB-A17, which potently neutralizes BA.1, uses IGKV3-20 and has an aa deletion along with an S31T SHM that, in combination, both shortens and stiffens the LCDR1, thereby reducing the entropic binding penalty while retaining suitable binding geometry in this highly mutated region of the Omicron RBD. The clonally related CAB-A18 does not have this shortening but carries other mutations in the LCDR1, indicating that multiple solutions exist. Interestingly, we find that SHMs in the light chain did not play a dominant role in the neutralization of BA.1 by CAB-A17.

In a relatively small set of antibodies from this public class, we identified three broadly cross-neutralizing antibodies from two clonally distinct lineages, suggesting that there are distinct ways to overcome mutations in the Omicron spike to achieve breadth and potency. Further, broad responses may be more generally achievable through affinity maturation than previously appreciated. This is supported by the discovery of similar antibodies in other individuals following Omicron breakthrough infection.[41]

Notably, the mAbs described here were isolated in Sweden in late 2020, before VoCs circulated.[42] This is encouraging, as it indicates that mutations selected for in the affinity maturation to the founder spike also afford improved cross-neutralization of Omicron. CAB-A17 and CAB-A18 breadth extended to include Omicron sublineages circulating 2.5 years after the initial infection that elicited the antibodies, representing a significant resistance to future escape.

The detection of cross-neutralizing antibodies within the repertoire of a convalescent individual suggests that such memory B cells are available to be recruited upon reinfection or vaccination. Indeed, such recruitment of memory B cells with enhanced potency and breadth has been observed following a third dose of the first-generation mRNA vaccines,[15,16] suggesting that affinity maturation broadened responses. Indeed, affinity maturation of antibody lineages occurs over the months after SARS-CoV-2 infection and is associated with the cross-neutralization of VoCs.[43] Here, we demonstrate that a common class of public antibodies can develop broad cross-neutralizing activity through affinity maturation. As a result, vaccination regimens tailored to drive the recruitment and affinity maturation of memory B cell responses could broaden protection against currently circulating and future variants.

Limitations of the study

We acknowledge that our conclusions regarding the role of SHM and affinity maturation in broadening antibody neutralization across SARS-CoV-2 variants are based on observed correlations. These interpretations would benefit from longitudinal studies tracking the evolution of antibody responses over extended time in individuals exposed to different viral strains. Furthermore, our findings, derived from a specific set of public antibodies, may not fully represent the diversity of immune responses across a wider population. The extent to which these antibodies are representative of the broader repertoire of human immune responses to SARS-CoV-2, particularly in diverse ethnic and genetic backgrounds, warrants further investigation.

STAR★METHODS

Detailed methods are provided in the online version of this paper and include the following:

- KEY RESOURCES TABLE
- RESOURCE AVAILABILITY
    - Lead contact
    - Materials availability
    - Data and code availability
- EXPERIMENTAL MODEL AND STUDY PARTICIPANT DETAILS
    - Sample collection
- METHOD DETAILS
    - Single-cell sorting of SARS-CoV-2-specific memory B cells by flow cytometry
    - Single B cell RT-PCR
    - Lineage phylogenetics
    - Cloning of engineered monoclonal antibodies
    - Expression and purification of engineered monoclonal antibodies
    - Cell culture
    - Spike expression vectors
    - Pseudovirus neutralization assay
    - Mutation frequencies
    - Preparation of fab fragments
    - Surface plasmon resonance assays
    - Cryo-EM sample preparation and imaging
    - Cryo-EM model building and structure refinement
    - FACS sorting of yeast libraries to select RBD mutants with reduced antibody binding
    - DNA extraction and illumina sequencing
- QUANTIFICATION AND STATISTICAL ANALYSIS
    - Analysis of deep sequencing data to compute each mutation's escape fraction
    - Statistical analysis

SUPPLEMENTAL INFORMATION

Supplemental information can be found online at https://doi.org/10.1016/j.xcrm.2024.101577.

ACKNOWLEDGMENTS

The pCMV-dR8.2 dvpr was a gift from Bob Weinberg (Addgene plasmid 8455; http://n2t.net/addgene:8455; RRID: Addgene_8455). pBOBI-FLuc (Addgene plasmid 170674; http://n2t.net/addgene:170674; RRID: Addgene_170674) and pcDNA3.3_CoV1_D28 (Addgene plasmid 170447; http://n2t.net/addgene:170447; RRID: Addgene_170447) were gifts from David Nemazee. We further acknowledge the Karolinska Institutet's 3D-EM facility, which was used to collect all cryo-EM data. Funding for this work was provided by grants from the Swedish Research Council to G.B.H.K. (2017-00968), B.M. (2018-02381), and B.M.H. (2017-6702, 2018-3808, and 2022-4822); the Knut and Alice Wallenberg Foundation to B.M.H.; the Erling Persson Foundation to G.B.K.H. and B.M.; the SciLifeLab Pandemic Laboratory Preparedness (PLP) Program 2022-2023 (VC-2022-0028) to G.B.K.H.; and the G2P-UK National Virology Consortium funded by MRC/UKRI (grant MR/W005611/1) to T.P.P.

AUTHOR CONTRIBUTIONS

Conceptualization, D.J.S., P.P., B.M., G.B.K.H., and B.M.H.; methodology, D.J.S., P.P., A.J.G., L.H., M.C., J.D.B., B.M., G.B.K.H., and B.M.H.; investigation, D.J.S., P.P., H.D., E.H., X.C.D., A.J.G., C.K., S.K., T.P.P., and B.M.H.; visualization, D.J.S., P.P., H.D., A.J.G., T.P.P., J.D.B., B.M., and B.M.H.; resources, L.H., R.D., G.M.M., and J.A.; funding acquisition, G.M.M., G.B.K.H., B.M., and B.M.H.; supervision, J.D.B., P.D., B.M., G.B.K.H., and B.M.H.; writing – original draft, D.J.S., B.M., G.B.K.H., and B.M.H.; writing – review & editing: D.J.S., P.P., H.D., A.J.G., J.A., B.M., G.B.K.H., and B.M.H.

DECLARATION OF INTERESTS

D.J.S. has consulted for AstraZeneca AB on topics related to viral evolution and the use of monoclonal antibodies for SARS-CoV-2. P.P. is currently employed by BioNTech. A.J.G. and J.D.B. have the potential to receive a share of IP revenue as inventors on the Fred Hutch-optioned technology related to deep mutational scanning of the receptor-binding domain of the SARS-CoV-2 spike protein. J.D.B. consults for Apriori Bio, Aerium Therapeutics, Invivyd, and the Vax Company on topics related to viral evolution. B.M.H. is founder and owner of Macrostruct Consulting AB. M.C. and G.B.K.H. are co-founders and co-owners of ImmuneDiscover Sweden AB.

Received: January 26, 2023
Revised: December 15, 2023
Accepted: April 24, 2024
Published: May 17, 2024

REFERENCES

1. Viana, R., Moyo, S., Amoako, D.G., Tegally, H., Scheepers, C., Althaus, C.L., Anyaneji, U.J., Bester, P.A., Boni, M.F., Chand, M., et al. (2022). Rapid epidemic expansion of the SARS-CoV-2 Omicron variant in

southern Africa. Nature *603*, 679–686. https://doi.org/10.1038/s41586-022-04411-y.

2. Planas, D., Saunders, N., Maes, P., Guivel-Benhassine, F., Planchais, C., Buchrieser, J., Bolland, W.-H., Porrot, F., Staropoli, I., Lemoine, F., et al. (2022). Considerable escape of SARS-CoV-2 Omicron to antibody neutralization. Nature *602*, 671–675. https://doi.org/10.1038/d41586-021-03827-2.
3. Cao, Y., Wang, J., Jian, F., Xiao, T., Song, W., Yisimayi, A., Huang, W., Li, Q., Wang, P., An, R., et al. (2022). Omicron escapes the majority of existing SARS-CoV-2 neutralizing antibodies. Nature *602*, 657–663. https://doi.org/10.1038/d41586-021-03796-6.
4. Yamasoba, D., Kosugi, Y., Kimura, I., Fujita, S., Uriu, K., Ito, J., and Sato, K.; Genotype to Phenotype Japan G2P-Japan Consortium (2022). Neutralisation sensitivity of SARS-CoV-2 omicron subvariants to therapeutic monoclonal antibodies. Lancet Infect. Dis. *22*, 942–943. https://doi.org/10.1016/S1473-3099(22)00365-6.
5. Cox, M., Peacock, T.P., Harvey, W.T., Hughes, J., Wright, D.W., Willett, B.J., Thomson, E., Gupta, R.K., Peacock, S.J., Robertson, D.L., and Carabelli, A.M. (2023). SARS-CoV-2 variant evasion of monoclonal antibodies based on in vitro studies. Nat. Rev. Microbiol. *21*, 112–124. https://doi.org/10.1038/s41579-022-00809-7.
6. Wang, Q., Guo, Y., Iketani, S., Nair, M.S., Li, Z., Mohri, H., Wang, M., Yu, J., Bowen, A.D., Chang, J.Y., et al. (2022). Antibody evasion by SARS-CoV-2 Omicron subvariants BA.2.12.1, BA.4 and BA.5. Nature *608*, 603–608. https://doi.org/10.1038/s41586-022-05053-w.
7. Sheward, D.J., Kim, C., Ehling, R.A., Pankow, A., Castro Dopico, X., Dyrdak, R., Martin, D.P., Reddy, S.T., Dillner, J., Karlsson Hedestam, G.B., et al. (2022). Neutralisation sensitivity of the SARS-CoV-2 omicron (B.1.1.529) variant: a cross-sectional study. Lancet Infect. Dis. *22*, 813–820. https://doi.org/10.1016/S1473-3099(22)00129-3.
8. Cele, S., Jackson, L., Khoury, D.S., Khan, K., Moyo-Gwete, T., Tegally, H., San, J.E., Cromer, D., Scheepers, C., Amoako, D.G., et al. (2022). Omicron extensively but incompletely escapes Pfizer BNT162b2 neutralization. Nature *602*, 654–656. https://doi.org/10.1038/s41586-021-04387-1.
9. Iketani, S., Liu, L., Guo, Y., Liu, L., Chan, J.F.-W., Huang, Y., Wang, M., Luo, Y., Yu, J., Chu, H., et al. (2022). Antibody evasion properties of SARS-CoV-2 Omicron sublineages. Nature *604*, 553–556. https://doi.org/10.1038/s41586-022-04594-4.
10. Sheward, D.J., Kim, C., Fischbach, J., Muschiol, S., Ehling, R.A., Björkström, N.K., Hedestam, G.B.K., Reddy, S.T., Albert, J., Peacock, T.P., et al. (2022). Evasion of neutralising antibodies by omicron sublineage BA.2.75. Lancet Infect. Dis., 1421–1422. https://doi.org/10.1016/S1473-3099(22)00524-2.
11. Cao, Y., Jian, F., Wang, J., Yu, Y., Song, W., Yisimayi, A., Wang, J., An, R., Chen, X., Zhang, N., et al. (2023). Imprinted SARS-CoV-2 humoral immunity induces convergent Omicron RBD evolution. Nature *614*, 521–529. https://doi.org/10.1038/s41586-022-05644-7.
12. Cao, Y., Yisimayi, A., Jian, F., Song, W., Xiao, T., Wang, L., Du, S., Wang, J., Li, Q., Chen, X., et al. (2022). BA.2.12.1, BA.4 and BA.5 escape antibodies elicited by Omicron infection. Nature *608*, 593–602. https://doi.org/10.1038/s41586-022-04980-y.
13. Sheward, D.J., Kim, C., Fischbach, J., Sato, K., Muschiol, S., Ehling, R.A., Björkström, N.K., Karlsson Hedestam, G.B., Reddy, S.T., Albert, J., et al. (2022). Omicron sublineage BA.2.75.2 exhibits extensive escape from neutralising antibodies. Lancet Infect. Dis. *22*, 1538–1540. https://doi.org/10.1016/S1473-3099(22)00663-6.
14. Kimura, I., Yamasoba, D., Tamura, T., Nao, N., Suzuki, T., Oda, Y., Mitoma, S., Ito, J., Nasser, H., Zahradnik, J., et al. (2022). Virological characteristics of the SARS-CoV-2 Omicron BA.2 subvariants, including BA.4 and BA.5. Cell *185*, 3992–4007.e16. https://doi.org/10.1016/j.cell.2022.09.018.
15. Garcia-Beltran, W.F., Denis, K.J.S., Hoelzemer, A., Lam, E.C., Nitido, A.D., Sheehan, M.L., Berrios, C., Ofoman, O., Chang, C.C., Hauser, B.M., et al. (2021). mRNA-based COVID-19 vaccine boosters induce neutralizing immunity against SARS-CoV-2 Omicron variant. Cell *185*, 457–466. https://doi.org/10.1016/j.cell.2021.12.033.
16. Muecksch, F., Wang, Z., Cho, A., Gaebler, C., Ben Tanfous, T., DaSilva, J., Bednarski, E., Ramos, V., Zong, S., Johnson, B., et al. (2022). Increased Memory B Cell Potency and Breadth After a SARS-CoV-2 mRNA Boost. Nature *607*, 128–134. https://doi.org/10.1038/s41586-022-04778-y.
17. Yuan, M., Liu, H., Wu, N.C., Lee, C.-C.D., Zhu, X., Zhao, F., Huang, D., Yu, W., Hua, Y., Tien, H., et al. (2020). Structural basis of a shared antibody response to SARS-CoV-2. Science *369*, 1119–1123. https://doi.org/10.1126/science.abd2321.
18. Tan, T.J.C., Yuan, M., Kuzelka, K., Padron, G.C., Beal, J.R., Chen, X., Wang, Y., Rivera-Cardona, J., Zhu, X., Stadtmueller, B.M., et al. (2021). Sequence signatures of two public antibody clonotypes that bind SARS-CoV-2 receptor binding domain. Nat. Commun. *12*, 3815. https://doi.org/10.1038/s41467-021-24123-7.
19. Barnes, C.O., Jette, C.A., Abernathy, M.E., Dam, K.-M.A., Esswein, S.R., Gristick, H.B., Malyutin, A.G., Sharaf, N.G., Huey-Tubman, K.E., Lee, Y.E., et al. (2020). SARS-CoV-2 neutralizing antibody structures inform therapeutic strategies. Nature *588*, 682–687. https://doi.org/10.1038/s41586-020-2852-1.
20. Dejnirattisai, W., Zhou, D., Supasa, P., Liu, C., Mentzer, A.J., Ginn, H.M., Zhao, Y., Duyvesteyn, H.M.E., Tuekprakhon, A., Nutalai, R., et al. (2021). Antibody evasion by the P.1 strain of SARS-CoV-2. Cell *184*, 2939–2954.e9. https://doi.org/10.1016/j.cell.2021.03.055.
21. Yuan, M., Huang, D., Lee, C.-C.D., Wu, N.C., Jackson, A.M., Zhu, X., Liu, H., Peng, L., van Gils, M.J., Sanders, R.W., et al. (2021). Structural and functional ramifications of antigenic drift in recent SARS-CoV-2 variants. Science *373*, 818–823. https://doi.org/10.1126/science.abh1139.
22. Pushparaj, P., Nicoletto, A., Sheward, D.J., Das, H., Castro Dopico, X., Perez Vidakovics, L., Hanke, L., Chernyshev, M., Narang, S., Kim, S., et al. (2023). Immunoglobulin germline gene polymorphisms influence the function of SARS-CoV-2 neutralizing antibodies. Immunity *56*, 193–206.e7. https://doi.org/10.1016/j.immuni.2022.12.005.
23. Barnes, C.O., West, A.P., Jr., Huey-Tubman, K.E., Hoffmann, M.A.G., Sharaf, N.G., Hoffman, P.R., Koranda, N., Gristick, H.B., Gaebler, C., Muecksch, F., et al. (2020). Structures of Human Antibodies Bound to SARS-CoV-2 Spike Reveal Common Epitopes and Recurrent Features of Antibodies. Cell *182*, 828–842.e16. https://doi.org/10.1016/j.cell.2020.06.025.
24. Starr, T.N., Greaney, A.J., Hilton, S.K., Ellis, D., Crawford, K.H.D., Dingens, A.S., Navarro, M.J., Bowen, J.E., Tortorici, M.A., Walls, A.C., et al. (2020). Deep Mutational Scanning of SARS-CoV-2 Receptor Binding Domain Reveals Constraints on Folding and ACE2 Binding. Cell *182*, 1295–1310.e20. https://doi.org/10.1016/j.cell.2020.08.012.
25. https://github.com/jbloomlab/SARS-CoV-2-RBD_MAP_Karolinska/blob/main/results/supp_data/karolinska_raw_data.csv
26. https://jbloomlab.github.io/SARS-CoV-2-RBD_MAP_Karolinska/
27. Greaney, A.J., Starr, T.N., Barnes, C.O., Weisblum, Y., Schmidt, F., Caskey, M., Gaebler, C., Cho, A., Agudelo, M., Finkin, S., et al. (2021). Mapping mutations to the SARS-CoV-2 RBD that escape binding by different classes of antibodies. Nat. Commun. *12*, 4196. https://doi.org/10.1038/s41467-021-24435-8.
28. Starr, T.N., Greaney, A.J., Addetia, A., Hannon, W.W., Choudhary, M.C., Dingens, A.S., Li, J.Z., and Bloom, J.D. (2021). Prospective mapping of viral mutations that escape antibodies used to treat COVID-19. Science *371*, 850–854. https://doi.org/10.1126/science.abf9302.
29. Zhang, Q., Ju, B., Ge, J., Chan, J.F.-W., Cheng, L., Wang, R., Huang, W., Fang, M., Chen, P., Zhou, B., et al. (2021). Potent and protective IGHV3-53/3-66 public antibodies and their shared escape mutant on the spike of SARS-CoV-2. Nat. Commun. *12*, 4210. https://doi.org/10.1038/s41467-021-24514-w.
30. Greaney, A.J., Starr, T.N., Gilchuk, P., Zost, S.J., Binshtein, E., Loes, A.N., Hilton, S.K., Huddleston, J., Eguia, R., Crawford, K.H.D., et al. (2021).

CellPress
OPEN ACCESS

Complete Mapping of Mutations to the SARS-CoV-2 Spike Receptor-Binding Domain that Escape Antibody Recognition. Cell Host Microbe *29*, 44–57.e9. https://doi.org/10.1016/j.chom.2020.11.007.

31. Gruell, H., Vanshylla, K., Tober-Lau, P., Hillus, D., Schommers, P., Lehmann, C., Kurth, F., Sander, L.E., and Klein, F. (2022). mRNA booster immunization elicits potent neutralizing serum activity against the SARS-CoV-2 Omicron variant. Nat. Med. *28*, 477–480. https://doi.org/10.1038/s41591-021-01676-0.
32. Teplyakov, A., Obmolova, G., Malia, T.J., Luo, J., Muzammil, S., Sweet, R., Almagro, J.C., and Gilliland, G.L. (2016). Structural diversity in a human antibody germline library. mAbs *8*, 1045–1063. https://doi.org/10.1080/19420862.2016.1190060.
33. Raybould, M.I.J., Kovaltsuk, A., Marks, C., and Deane, C.M. (2021). CoV-AbDab: the coronavirus antibody database. Bioinformatics *37*, 734–735. https://doi.org/10.1093/bioinformatics/btaa739.
34. Westendorf, K., Žentelis, S., Wang, L., Foster, D., Vaillancourt, P., Wiggin, M., Lovett, E., van der Lee, R., Hendle, J., Pustilnik, A., et al. (2022). LY-CoV1404 (bebtelovimab) potently neutralizes SARS-CoV-2 variants. Cell Rep. *39*, 110812. https://doi.org/10.1016/j.celrep.2022.110812.
35. Rogers, T.F., Zhao, F., Huang, D., Beutler, N., Burns, A., He, W.-T., Limbo, O., Smith, C., Song, G., Woehl, J., et al. (2020). Isolation of potent SARS-CoV-2 neutralizing antibodies and protection from disease in a small animal model. Science *369*, 956–963. https://doi.org/10.1126/science.abc7520.
36. Robbiani, D.F., Gaebler, C., Muecksch, F., Lorenzi, J.C.C., Wang, Z., Cho, A., Agudelo, M., Barnes, C.O., Gazumyan, A., Finkin, S., et al. (2020). Convergent antibody responses to SARS-CoV-2 in convalescent individuals. Nature *584*, 437–442. https://doi.org/10.1038/s41586-020-2456-9.
37. Cao, Y., Su, B., Guo, X., Sun, W., Deng, Y., Bao, L., Zhu, Q., Zhang, X., Zheng, Y., Geng, C., et al. (2020). Potent Neutralizing Antibodies against SARS-CoV-2 Identified by High-Throughput Single-Cell Sequencing of Convalescent Patients' B Cells. Cell *182*, 73–84.e16. https://doi.org/10.1016/j.cell.2020.05.025.
38. Banach, B.B., Cerutti, G., Fahad, A.S., Shen, C.-H., Oliveira De Souza, M., Katsamba, P.S., Tsybovsky, Y., Wang, P., Nair, M.S., Huang, Y., et al. (2021). Paired heavy- and light-chain signatures contribute to potent SARS-CoV-2 neutralization in public antibody responses. Cell Rep. *37*, 109771. https://doi.org/10.1016/j.celrep.2021.109771.
39. Thorpe, I.F., and Brooks, C.L. (2007). Molecular evolution of affinity and flexibility in the immune system. Proc. Natl. Acad. Sci. USA *104*, 8821–8826. https://doi.org/10.1073/pnas.0610064104.
40. Wang, M., Fan, Q., Zhou, B., Ye, H., Shen, S., Yu, J., Cheng, L., Ge, X., Ju, B., and Zhang, Z. (2022). A key F27I substitution within HCDR1 facilitates the rapid maturation of P2C-1F11-like neutralizing antibodies in a SARS-CoV-2-infected donor. Cell Rep. *40*, 111335. https://doi.org/10.1016/j.celrep.2022.111335.
41. Nutalai, R., Zhou, D., Tuekprakhon, A., Ginn, H.M., Supasa, P., Liu, C., Huo, J., Mentzer, A.J., Duyvesteyn, H.M.E., Dijokaite-Guraliuc, A., et al. (2022). Potent cross-reactive antibodies following Omicron breakthrough in vaccinees. Cell *185*, 2116–2131.e18. https://doi.org/10.1016/j.cell.2022.05.014.
42. Sheward, D.J., Mandolesi, M., Urgard, E., Kim, C., Hanke, L., Perez Vidakovics, L., Pankow, A., Smith, N.L., Castro Dopico, X., McInerney, G.M., et al. (2021). Beta RBD boost broadens antibody-mediated protection against SARS-CoV-2 variants in animal models. Cell Rep. Med. *2*, 100450. https://doi.org/10.1016/j.xcrm.2021.100450.
43. Muecksch, F., Weisblum, Y., Barnes, C.O., Schmidt, F., Schaefer-Babajew, D., Wang, Z., C Lorenzi, J.C., Flyak, A.I., DeLaitsch, A.T., Huey-Tubman, K.E., et al. (2021). Affinity maturation of SARS-CoV-2 neutralizing antibodies confers potency, breadth, and resilience to viral escape mutations. Immunity *54*, 1853–1868.e7. https://doi.org/10.1016/j.immuni.2021.07.008.
44. Hanke, L., Vidakovics Perez, L., Sheward, D.J., Das, H., Schulte, T., Moliner-Morro, A., Corcoran, M., Achour, A., Karlsson Hedestam, G.B., Hällberg, B.M., et al. (2020). An alpaca nanobody neutralizes SARS- CoV-2 by blocking receptor interaction. Nat. Commun. *11*, 4420. https://doi.org/10.1038/s41467-020-18174-5.
45. Vázquez Bernat, N., Corcoran, M., Hardt, U., Kaduk, M., Phad, G.E., Martin, M., and Karlsson Hedestam, G.B. (2019). High-quality library preparation for NGS-based immunoglobulin germline gene inference and repertoire expression analysis. Front. Immunol. *10*, 660. https://doi.org/10.3389/fimmu.2019.00660.
46. Tiller, T., Meffre, E., Yurasov, S., Tsuiji, M., Nussenzweig, M.C., and Wardemann, H. (2008). Efficient generation of monoclonal antibodies from single human B cells by single cell RT-PCR and expression vector cloning. J. Immunol. Methods *329*, 112–124. https://doi.org/10.1016/j. jim.2007.09.017.
47. Stewart, S.A., Dykxhoorn, D.M., Palliser, D., Mizuno, H., Yu, E.Y., An, D.S., Sabatini, D.M., Chen, I.S.Y., Hahn, W.C., Sharp, P.A., et al. (2003). Lentivirus-delivered stable gene silencing by RNAi in primary cells. RNA *9*, 493–501. https://doi.org/10.1261/rna.2192803.
48. Tegunov, D., and Cramer, P. (2019). Real-time cryo-electron microscopy data preprocessing with Warp. Nat. Methods *16*, 1146–1152. https://doi.org/10.1038/s41592-019-0580-y.
49. Punjani, A., Rubinstein, J.L., Fleet, D.J., and Brubaker, M.A. (2017). cryoSPARC: algorithms for rapid unsupervised cryo-EM structure determination. Nat. Methods *14*, 290–296. https://doi.org/10.1038/nmeth.4169.
50. Emsley, P., Lohkamp, B., Scott, W.G., and Cowtan, K. (2010). Features and development of Coot. Acta Crystallogr. D Biol. Crystallogr. *66*, 486–501. https://doi.org/10.1107/S0907444910007493.
51. Goddard, T.D., Huang, C.C., Meng, E.C., Pettersen, E.F., Couch, G.S., Morris, J.H., and Ferrin, T.E. (2018). UCSF ChimeraX: Meeting modern challenges in visualization and analysis. Protein Sci. *27*, 14–25. https://doi.org/10.1002/pro.3235.
52. Adams, P.D., Afonine, P.V., Bunkóczi, G., Chen, V.B., Davis, I.W., Echols, N., Headd, J.J., Hung, L.-W., Kapral, G.J., Grosse-Kunstleve, R.W., et al. (2010). PHENIX: a comprehensive Python-based system for macromolecular structure solution. Acta Crystallogr. D Biol. Crystallogr. *66*, 213–221. https://doi.org/10.1107/S0907444909052925.
53. Price, M.N., Dehal, P.S., and Arkin, A.P. (2010). FastTree 2 - Approximately Maximum-Likelihood Trees for Large Alignments. Plos One *5*, e9040. https://doi.org/10.1371/journal.pone.0009490.
54. Hsieh, C.-L., Goldsmith, J.A., Schaub, J.M., DiVenere, A.M., Kuo, H.-C., Javanmardi, K., Le, K.C., Wrapp, D., Lee, A.G., Liu, Y., et al. (2020). Structure-based design of prefusion-stabilized SARS-CoV-2 spikes. Science *369*, 1501–1505. https://doi.org/10.1126/science.abd0826.
55. Custódio, T.F., Das, H., Sheward, D.J., Hanke, L., Pazicky, S., Pieprzyk, J., Sorgenfrei, M., Schroer, M.A., Gruzinov, A.Y., Jeffries, C.M., et al. (2020). Selection, biophysical and structural analysis of synthetic nanobodies that effectively neutralize SARS-CoV-2. Nat. Commun. *11*, 5588. https://doi.org/10.1038/s41467-020-19204-y.
56. Williams, C.J., Headd, J.J., Moriarty, N.W., Prisant, M.G., Videau, L.L., Deis, L.N., Verma, V., Keedy, D.A., Hintze, B.J., Chen, V.B., et al. (2018). MolProbity: More and better reference data for improved all-atom structure validation. Protein Sci. *27*, 293–315. https://doi.org/10.1002/pro.3330.
57. Elbe, S., and Buckland-Merrett, G. (2017). Data, disease and diplomacy: GISAID's innovative contribution to global health. Glob. Chall. *1*, 33–46. https://doi.org/10.1002/gch2.1018.

STAR★METHODS

KEY RESOURCES TABLE

REAGENT or RESOURCE	SOURCE	IDENTIFIER
Antibodies		
Anti-C-Myc Antibody (Chicken) - FITC Conjugated	Immunology Consultants Laboratory, Inc.	Cat# CMYC-45F
Allophycocyanin (APC) AffiniPure Goat Anti-Human IgG, Fcγ fragment specific	Jackson ImmunoResearch	Cat#109-135-098; RRID: AB_2337690
Goat Anti-Human IgG(H + L)-HRP	Southern Biotech	Cat#2015-05; RRID:AB_2795588
BD Pharmingen FITC Mouse Anti-Human CD3ε	BD Bioscience	Cat#556611; RRID:AB_396484
BD Pharmingen FITC Mouse Anti-Human CD14	BD Bioscience	Cat#557153; RRID:AB_396589
BD Horizon BV421 Mouse Anti-Human CD20	BD Bioscience	Cat#562873; RRID:AB_2737857
BD Pharmingen PE-Cy7 Mouse Anti-Human CD27	BD Bioscience	Cat#560609; RRID:AB_1727456
BD Horizon PE-CF594 Mouse Anti-Human IgG	BD Bioscience	Cat#562538; RRID:AB_2737640
Fc Receptor Binding Inhibitor Polyclonal Antibody	Thermo Fisher	Cat#14-9161-73; RRID:AB_468582
Bacterial and virus strains		
E. coli XL10-Gold Ultracompetent Cells	Agilent Technologies	Cat#200315
Chemicals, peptides, and recombinant proteins		
Stabilized Soluble Spike	Pushparaj et al.[22]	N/A
Biotinylated RBD	Acro Biosystems	SPD-C82E4
Stabilized soluble Omicron BA.1 spike	Acro Biosystems	SPN-C82Ee
FreeStyle™ 293 Expression Medium	Thermo Fisher	Cat#12338018
FreeStyle™ MAX Reagent	Thermo Fisher	Cat#16447100
Lipofectamine 3000	Invitrogen	Cat# L3000075
Critical commercial assays		
Superscript IV RT kit	Invitrogen	Cat#18090010
HotStarTaq Plus Kit	Qiagen	Cat#203203
Gibson Assembly Master Mix	New England Biolabs	Cat#E2611L
QIAGEN Plasmid Plus Midi Kit	Qiagen	Cat#12945
Pierce Fab Preparation Kit	Thermo Fisher	Cat#44985
LIVE/DEAD™ Fixable Aqua Dead Cell Stain Kit	Thermo Fisher	Cat#L34957
Bright-Glo Luciferase Assay System	Promega	Cat#E2620
Sensor Chip SA	Cytiva	BR100398
Zymoprep Yeast Plasmid Miniprep II	Zymo Research	Cat#D2004
Deposited data		
V(D)Js of neutralizing IgGs HC and LCs	Pushparaj et al.[22]	GenBank accession numbers ON086918-25, ON086933-40
Omicron spike + Fab fragment of CAB-A17	This paper	PDB: 8C2R; EMD: EMD-16397
Localized reconstruction of Omicron RBD + Fab fragment of CAB-A17	This paper	PDB: 8V4F; EMD: EMD-42970
Experimental models: Cell lines		
FreeStyle™ 293-F Cells	Thermo Fisher	Cat#R79007
HEK293T-ACE2	Hanke et al.[44]	N/A
Oligonucleotides		
Colony PCR primers	Vázquez Bernat et al.[45]	N/A
Recombinant DNA		
AbVec2.0-IGHG1	Tiller et al.[46]	Addgene 80795; RRID: Addgene_80795
AbVec1.1-IGKC	Tiller et al.[46]	Addgene 80796; RRID:Addgene_80796

(*Continued on next page*)

Continued

REAGENT or RESOURCE	SOURCE	IDENTIFIER
AbVec1.1-IGLC2-XhoI	Tiller et al.[46]	Addgene 99575; RRID:Addgene_99575
pCMV-dR8.2 dvpr	Stewart et al.[47]	Addgene 8455; RRID:Addgene_8455
pBOBI-FLuc	Rogers et al.[35]	Addgene 170674; RRID:Addgene_170674
Software and algorithms		
GraphPad Prism, version 9	GraphPad Software	https://www.graphpad.com
EPU	Thermo Fisher Scientific	https://www.thermofisher.com/se/en/home/electron-microscopy/products/software-em-3d-vis/epu-software.html
Warp	Tegunov and Cramer[48]	http://www.warpem.com/warp/?page_id=65
cryoSPARC v.3.3.1	Punjani et al.[49]	https://cryosparc.com/
Coot	Emsley et al.[50]	https://www2.mrc-lmb.cam.ac.uk/personal/pemsley/coot/
ChimeraX	Goddard et al.[51]	https://www.rbvi.ucsf.edu/chimerax/
PHENIX 1.19.2	Adams et al.[52]	https://phenix-online.org/documentation/index.html
dms_variants package version 0.8.10	Starr et al.[24]	https://jbloomlab.github.io/dms_variants/
Full workflow used for the DMS analysis in this paper	Starr et al.[24] and this work	https://doi.org/10.5281/zenodo.11008143
Other		
NuPAGE 4 to 12%, Bis-Tris, 1.0 mm, Mini Protein Gel, 12-well	Thermo Fisher	Cat#NP0322BOX
GloMax Navigator Microplate Luminometer	Promega	Cat#GM2000
CryoMatrix® holey grids (R 2/1 geometry)	Zhenjiang Lehua Technology Co., Ltd	M025-Au300-R20/10
Vitrobot Mk IV	Thermo Fisher Scientific	N/A
EMS 100X	Electron Microscopy Sciences	N/A
Vitrobot filter paper 595	Ted Pella	47000-100
Krios G3i	Thermo Fisher Scientific	N/A
BioQuantum K3	Gatan	1967
Protein G Sepharose columns	Cytiva	29-0588-06

RESOURCE AVAILABILITY

Lead contact

Further information and requests for resources and reagents should be directed to and will be fulfilled by the lead contact, Martin Hällberg (martin.hallberg@ki.se).

Materials availability

Plasmids and Cell lines used in this study can be made available under an appropriate materials transfer agreement. No other unique reagents were generated.

Data and code availability

- Data: The cryo-EM reconstructions have been deposited in the Electron Microscopy DataBank under accession codes EMD-16397 (Omicron spike + Fab fragment of CAB-A17), EMD-42970 (localized reconstruction of Omicron RBD + Fab fragment of CAB-A17). The atomic coordinates have been deposited in the Protein DataBank under IDs 8C2R (Omicron spike + Fab fragment of CAB-A17), 8V4F (localized reconstruction of Omicron RBD + Fab fragment of CAB-A17). Nucleotide sequences for antibodies described in this publication are available at GenBank, with accessions ON086918-ON086925 (HCs) and ON086933-ON086940 (LCs). All other datasets generated during and/or analyzed during the current study are available from the corresponding authors. The raw Illumina FASTQ files for the yeast-displayed antibody-escape experiments are available on the NCBI Short Read Archive (SRA) under BioProject PRJNA639956, BioSample SAMN26873633. The complete antibody-escape scores are available in[25] and at https://doi.org/10.5281/zenodo.11008143.

- Code: This study does not generate custom code but full documentation on how the analysis was performed is available at https://doi.org/10.5281/zenodo.11008143.
- Any additional information required to reanalyze the data reported in this work paper is available from the lead contact upon request.

EXPERIMENTAL MODEL AND STUDY PARTICIPANT DETAILS

Sample collection

Hospital workers at the Karolinska University Hospital in Stockholm, Sweden, were invited to participate in a study to characterize their antibody responses following SARS-CoV-2 infection. Participants who were confirmed PCR-positive in May 2020 provided blood samples in December 2020. Informed consent was obtained from all participants as part of ethics approvals (Decisions# 2020-01620, 2020–02881, and 2020–05630) from the National Ethical Review Agency of Sweden. Blood was collected in EDTA tubes to separate plasma and peripheral blood mononuclear cells (PBMCs). PBMC isolation was performed by density-gradient centrifugation using Ficoll-Paque PLUS (GE Healthcare) or SepMate PBMC Isolation tubes (Stem Cell technologies). After a gradient separation, the mononuclear cell layers were collected, and the cell pellets were washed with sterile PBS. The isolated PBMCs were frozen in FBS with 10% dimethyl sulfoxide (DMSO) (Sigma).

METHOD DETAILS

Single-cell sorting of SARS-CoV-2-specific memory B cells by flow cytometry

Spike-specific memory B cells were stained and single-cell-sorted from PBMCs using a three-laser FACSAria cell sorter (BD Bioscience) by gating on live, $CD3^{-}$, $CD14^{-}$, $CD20^{+}$, $CD27^{+}$, IgG+, spike+ cells into 96-well PCR plates containing 4 μL of cell lysis buffer. All fluorescently labeled antibodies and the biotinylated spike-trimer conjugated to streptavidin-allophycocyanin (SA-APC) (Invitrogen) were titrated before sorting. Plates were sealed, frozen on dry ice, and stored at −80°C.

Single B cell RT-PCR

The 96-well plates containing lysed single B cells were thawed on ice, and cDNA was generated by reverse transcription using random hexamers, dNTPs, and SuperScript IV reverse transcriptase (Invitrogen). V(D)J sequences were amplified separately in 25 μL nested PCR reactions using 3 μL of cDNA in the 1st round PCR and 1.5 μL PCR product in the 2nd round PCR. The HotStarTaq Plus Kit (Qiagen) and 5′-leader sequence-specific and 3′-IgG-specific primers were used. PCR products from positive wells were purified, Sanger sequenced (Genewiz), and analyzed.

Lineage phylogenetics

A phylogeny was inferred from the heavy chain CAB-A17, CAB-A18, and CAB-A49 sequences, rooted using the inferred germline V and J genes, using FastTree,[53] compiled with the double-precision flag, and using a GTR nucleotide model.

Cloning of engineered monoclonal antibodies

The back mutated HC and LC sequences were designed appropriately with overhangs that match the ends of the linearized vector and ordered from Integrated DNA Technologies. The ordered inserts were cloned into expression vectors containing human Igγ1 H, Igκ1 L, or Igλ2 L constant regions by Gibson assembly. Gibson assembly was performed using a Gibson master mix (New England Biolabs), 50ng of vector, 30 ng of insert in a 20 μL reaction, incubated at 50°C for 1 h, and transformed into XL10-Gold ultracompetent cells by heat shock at 42°C for 30 s, according to the manufacturer's protocol (Agilent Technologies). Colonies were screened[45] by PCR and confirmed by Sanger sequencing (Genewiz). Positive colonies were expanded, and plasmids were purified using a plasmid Midi-prep kit (Qiagen) per the manufacturer's instructions.

Expression and purification of engineered monoclonal antibodies

For mAb expression, 15 μg of each HC and LC vector DNA was transfected into FreeStyle 293-F cells, cultured in 30 mL of FreeStyle 293 expression medium (Life Technologies) at a cell density of 1×10^6 cells/mL and ≥90% viability, using 30 μL of freestyle max reagent (Life Technologies) according to the manufacturer's protocol. mAbs were harvested and purified seven days after transfection using Protein G Sepharose columns (GE Healthcare). All purified recombinant mAbs were analyzed by SDS-PAGE under reducing conditions using NuPAGE Novex 4–12% Bis-Tris polyacrylamide gels and NuPAGE reducing agent (Life Technologies) according to the manufacturer's instructions and by binding to SARS-CoV-2 spike and RBD by ELISA.

Cell culture

HEK293T cells (ATCC CRL-3216) and HEK293T-ACE2 cells (stably expressing human ACE2) were cultured in Dulbecco's Modified Eagle Medium (high glucose, with sodium pyruvate) supplemented with 10% fetal calf serum, 100 units/mL penicillin, and 100 μg/mL streptomycin. Cultures were maintained in a humidified 37°C incubator (5% CO_2).

Spike expression vectors

Spike expression vectors for pseudovirus neutralization assays encoded the variant spikes with the last 19 amino acids (or 28 amino acids in the case of SARS-CoV) truncated to enhance incorporation. Spike variants were generated by multi-site directed mutagenesis, and all mutated plasmids were subsequently confirmed by Sanger sequencing. The SARS-CoV spike expression vector was a gift from David Nemazee (Addgene #170447).

Pseudovirus neutralization assay

The co-transfection of HEK293T cells generated spike-pseudotyped lentivirus particles with a spike-encoding plasmid, an HIV gag-pol packaging plasmid (Addgene #8455), and a lentiviral transfer plasmid encoding firefly luciferase (Addgene #170674) using polyethyleneimine (PEI). Pseudoviruses were harvested from the supernatant 48- and 72-h post-transfection, clarified by centrifugation, and stored at −80°C until use. Pseudovirus neutralization assays were performed using HEK293T-ACE2 cells, as previously described.[42] Briefly, pseudoviruses titrated to produce ~100,000 RLUs were incubated with serially diluted mAbs for 60 min at 37°C in black-walled 96-well plates. 10,000 HEK293T-ACE2 cells[44] were then added to each well, and plates were incubated for 44–48 h. Luminescence was measured using Bright-Glo (Promega) on a GloMax Navigator Luminometer (Promega). Neutralization was calculated relative to the average of 8 control wells infected in the absence of serum. All fold-change estimates comparisons used IC_{50} values from neutralization assays run side-by-side.

Mutation frequencies

From the Coronavirus-Binding Antibody Sequences & Structures (CoV-AbDab) database,[33] we identified 162 anti-SARS-CoV-2 RBD monoclonal antibodies of human origin, used V3-53 or V3-66 germlines, had a CDR3 length <13, and used either V3-20, V1-9, or V1-33 light chain V genes.

Preparation of fab fragments

Fab fragments were prepared by digesting IgG with immobilized papain and separation of Fab and Fc fragments with a Protein A column, using the Pierce Fab Preparation Kit (ThermoFisher Scientific) per the manufacturer's instructions.

Surface plasmon resonance assays

Surface plasmon resonance was measured using a Biacore 2000 system with HBS running buffer (10 mM HEPES, 150 mM NaCl, 0.005% surfactant Tween 20 at pH 7.4). Site-specific biotinylated RBD-protein (Acro Biosystems) was immobilized to ~100 resonance units (RU) on streptavidin surface Sensor Chip SA (Cytiva). MAbs were injected at 30 μL/min (180-s association, 900-s dissociation). Surface regeneration was performed with 10 mM glycine-HCL buffer pH 1.5 for 2 × 20 s. Data processing and overlay plots were generated with BIA evaluation software, and curve fitting was performed using a bivalent model.

Cryo-EM sample preparation and imaging

Spike trimer (1.5 mg/mL; B.1.1.529 Omicron spike including prefusion stabilizing HexaPro mutations[54]; Acro Biosciences) and a Fab preparation of CAB-A17 were mixed in a 1:0.8 M ratio (S protein monomer–Fab), followed by incubation on ice for 20 min. Prior to cryo-EM grid preparation, grids were glow-discharged with 25 mA for 2 min using an EMS 100X (Electron Microscopy Sciences) glow-discharge unit. The grids used were CryoMatrix holey grids with amorphous alloy film (Nitinol; R 2/1 geometry; Zhenjiang Lehua Technology Co., Ltd). 3 μL aliquots of Fab preparations were applied to the grids; grids were then hand-blotted from the side, followed by two applications of the pre-incubated Omicron-Fab mixture with intermittent hand-blotting. Finally, the grids with sample were vitrified in a Vitrobot Mk IV (Thermo Fisher Scientific) at 4°C and 100% humidity [blot 11 s, blot force 4, 595 filter paper (Ted Pella Inc.)].

Cryo-EM data collection was performed with EPU 2.13 (Thermo Fisher Scientific) using a Krios G3i transmission-electron microscope (Thermo Fisher Scientific) operated at 300 kV in the Karolinska Institutet's 3D-EM facility. Movies were acquired in nanoprobe EFTEM SA mode at 165 kx nominal magnification with a slit width of 10 eV using a K3 Bioquantum (operated in CDS mode) for 2 s, during which 60 movie frames were collected with a fluence of 0.91 $e^{-}/Å^{2}$ per frame (see Table S1). A stage tilt of 20-degrees was used to alleviate preferred particle orientations. Motion correction, and Fourier cropping (to 1.01 Å per pixel), were performed on the fly using Warp.[48] Micrographs were imported into CryoSPARC 3.31,[49] and CTF parameters were estimated using a patch model.

A total of 19,561 micrographs were selected on the basis of an estimated resolution cutoff of 6 Å and defocus below 4 microns. Particles were picked in CryoSPARC through template matching using projections generated from a previous reconstruction of the ancestral SARS-CoV-2 spike. Extracted particles were used for a four-class ab-initio model generation followed by three rounds of heterogeneous refinement in CryoSPARC with an ensuing nonuniform 3D refinement of the resulting particles for the spike-containing particle set. Local CTF refinements were performed interspersed with global aberration estimation and correction (beam tilt, trefoil, tetrafoil, and anisotropic magnification). The two bound Fabs of CAB-A17 had varying orientations relative to the body of the spike and relative to each other. Therefore, 3D classification was performed within CryoSPARC 3.31 without pose alignment using 20 classes with a mask around the two RBDs in up conformations and their bound Fabs. One class was selected for further processing, and from the particles (199,751 out of a total of 398,386) belonging to this class, we performed local reconstruction of the volume close to one of the RBD-Fab interfaces. This process significantly enhanced the resolvability of the map and thereby enabled

molecular fitting and interpretation of the maps (Figure S3). All particles were processed with C1 symmetry. Please see Table S1 for data collection and processing statistics.

Cryo-EM model building and structure refinement

The structure of the ancestral spike protein trimer in 2-up conformation PDB: 7A29 [55] was used as a starting model for model building. The model was mutated and rebuilt manually to reflect the Omicron spike sequence and structure. Manual model building and structure refinement were performed using COOT[50] and PHENIX,[52] respectively, in interspersed cycles with secondary structure, Ramachandran, rotamers, and bond geometry restraints. Structure figures and EM density-map figures were generated with PyMol (Schrödinger), UCSF ChimeraX,[51] and COOT. All validation was performed with MolProbity.[56] Please see Table S1 for refinement and validation results.

FACS sorting of yeast libraries to select RBD mutants with reduced antibody binding

Antibody mapping experiments were performed in biological duplicates using the independent mutant RBD libraries, as previously described.[28,30] Mutant yeast libraries pre-sorted for RBD expression and ACE2 binding[30] induced to express RBD were washed and incubated with the antibody at the IC90 for binding to the unmutated Wuhan-Hu-1 RBD as measured by flow cytometry for 1 h at room temperature with gentle agitation. After the plasma incubations, the libraries were secondarily labeled for 1 h with 1:100 fluorescein isothiocyanate-conjugated anti-MYC antibody (Immunology Consultants Lab, CYMC-45F) to label for RBD expression and 1:200 Alexa Fluor-647-conjugated goat anti-human-IgG Fc-gamma (Jackson ImmunoResearch 109-135-098) to label for bound antibody. A flow cytometric selection gate was drawn to capture RBD mutants with reduced antibody binding for their degree of RBD expression (Figure S2). Approximately 107 cells were processed for each sample on the BD FACSAria II cell sorter. Antibody-escaped cells were grown overnight in synthetic defined medium with casamino acids (6.7 g/L Yeast Nitrogen Base, 5.0 g/L Casamino acids, 1.065 g/L MES acid, and 2% w/v dextrose +100 U/mL penicillin + 100 μg/mL streptomycin) to expand cells before plasmid extraction.

DNA extraction and illumina sequencing

Plasmid samples were prepared from 30 optical density (OD) units (1.6e8 colony forming units (cfus)) of pre-selection yeast populations and approximately 5 OD units (~3.2e7 cfus) of overnight cultures of plasma-escaped cells (Zymoprep Yeast Plasmid Miniprep II) as previously described.[30,57] The 16-nucleotide barcode sequences identifying each RBD variant were amplified by polymerase chain reaction (PCR) and prepared for Illumina sequencing as described.[30,57] Barcodes were sequenced on an Illumina NextSeq 2000 with 50 bp single-end reads.

QUANTIFICATION AND STATISTICAL ANALYSIS

Analysis of deep sequencing data to compute each mutation's escape fraction

Escape fractions were computed essentially as described.[28] We used the dms_variants package (https://jbloomlab.github.io/dms_variants/, version 0.8.10) to process Illumina sequences into counts of each barcoded RBD variant in each pre-selection and antibody-escape population. For each plasma selection, we computed the escape fraction for each barcoded variant using the deep sequencing counts for each variant in the original and plasma-escape populations and the total fraction of the library that escaped antibody binding via the formula provided in Greaney et al.[30] These escape fractions represent the estimated fraction of cells expressing that specific variant that falls in the escape bin, such that a value of 0 means the variant is always bound by plasma and a value of 1 means that it always escapes plasma binding.

We then applied a computational filter to remove variants with >1 amino acid mutation, low sequencing counts, or highly deleterious mutations that might cause antibody escape simply by leading to poor expression of properly folded RBD on the yeast cell surface as described.[28] Specifically, we removed mutations with an ACE2 binding score < -2.35 or RBD expression score < -1.0.[57] The reported antibody-escape scores throughout the paper are the average across the libraries with the correlations in final single-mutant escape scores are shown in Figure S2E. Full documentation of the computational analysis at[26] and at https://doi.org/10.5281/zenodo.11008143.

Statistical analysis

Neutralizing IC_{50} values were calculated in Prism v9 (GraphPad Software) by fitting a four-parameter logistic curve, to neutralization by serial 3-fold dilutions. Neutralization was bounded between 0 and 100%.

Cell Reports Medicine, Volume *5*

Supplemental information

Structural basis of broad SARS-CoV-2 cross-neutralization by affinity-matured public antibodies

Daniel J. Sheward, Pradeepa Pushparaj, Hrishikesh Das, Allison J. Greaney, Changil Kim, Sungyong Kim, Leo Hanke, Erik Hyllner, Robert Dyrdak, Jimin Lee, Xaquin Castro Dopico, Pia Dosenovic, Thomas P. Peacock, Gerald M. McInerney, Jan Albert, Martin Corcoran, Jesse D. Bloom, Ben Murrell, Gunilla B. Karlsson Hedestam, and B. Martin Hällberg

Cell Reports Medicine

Structural basis of broad SARS-CoV-2 cross-neutralization by affinity-matured public antibodies

Daniel J. Sheward, Pradeepa Pushparaj, Hrishikesh Das, Allison J. Greaney, Changil Kim, Sungyong Kim, Leo Hanke, Erik Hyllner, Robert Dyrdak, Jimin Lee, Xaquin Castro Dopico, Pia Dosenovic, Thomas P. Peacock, Gerald M. McInerney, Jan Albert, Martin Corcoran, Jesse D. Bloom, Ben Murrell, Gunilla B. Karlsson Hedestam, and B. Martin Hällberg

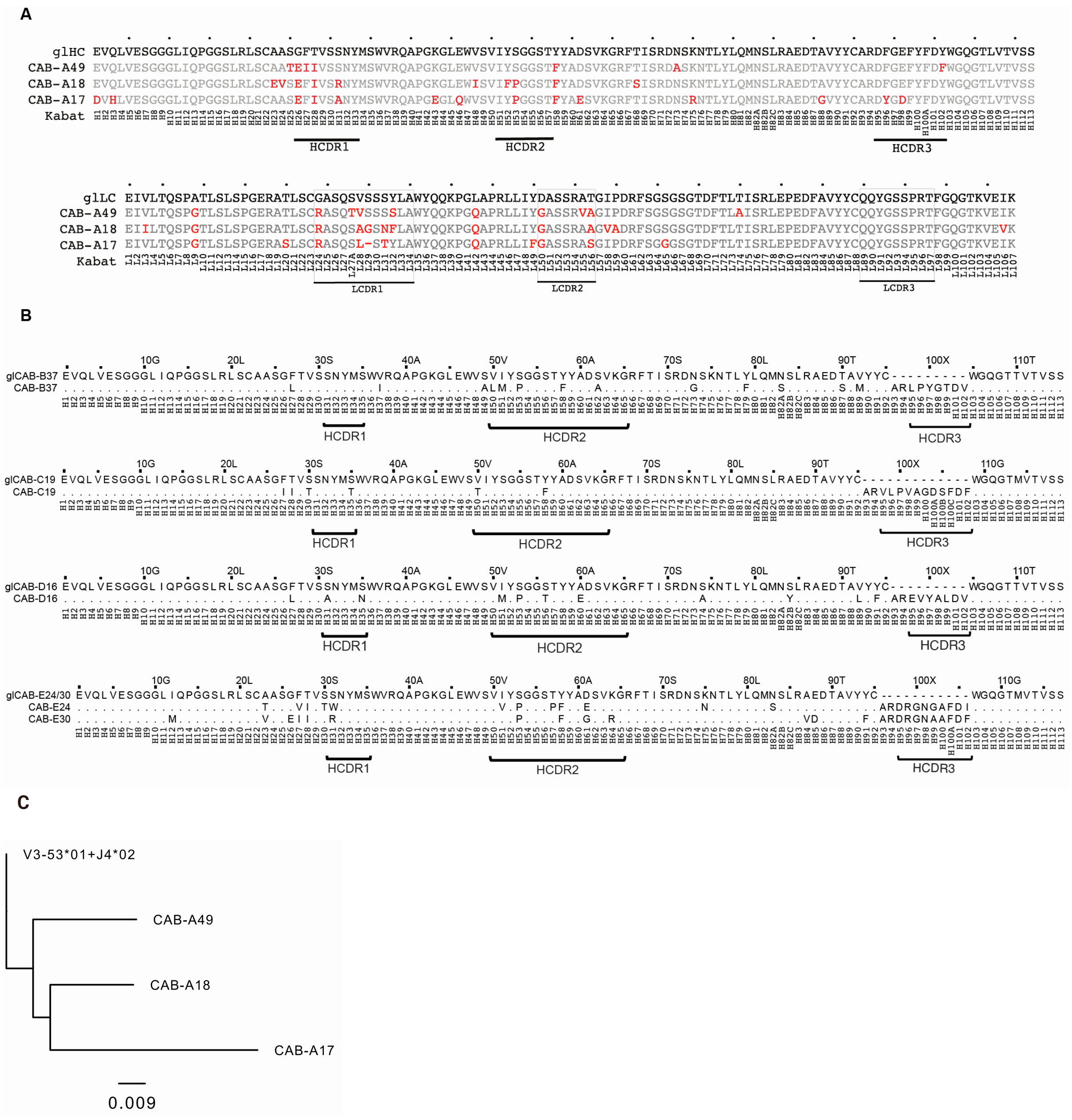

Figure S1. Alignment of sequences for the four lineages studied in this work. Related to Figure 1. **(A)** Alignment of heavy- and light-chain sequences of mAbs from lineage CAB-A. CDR borders and numbering are shown according to the Kabat scheme. Somatic hypermutations away from the germline (gl) are shown in red. **(B)** Alignment of heavy-chain sequences with their inferred germline for antibody lineages B-E. **(C)** A phylogeny of the CAB-A lineage, inferred from the heavy chain CAB-A17, CAB-A18, and CAB-A49 sequences, rooted on the inferred germline V and J genes.

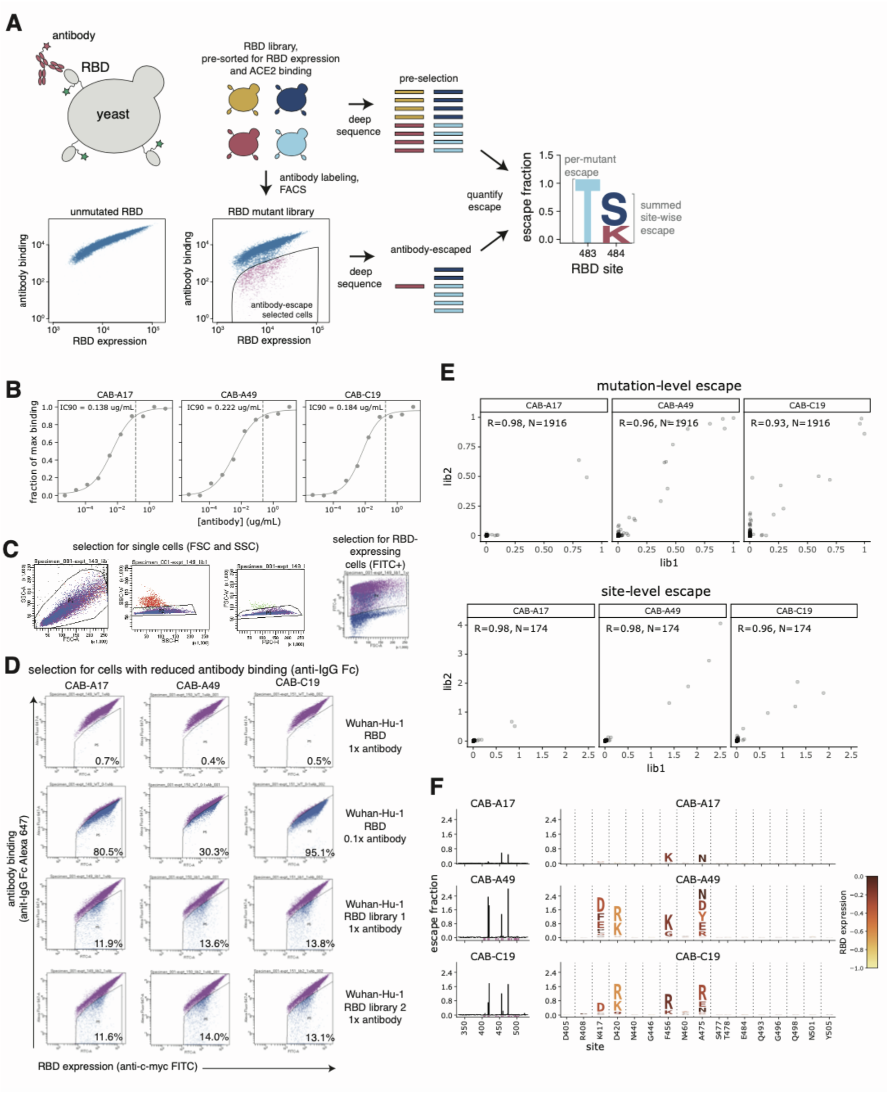

Figure S2. Complete mapping of mutations that escape antibody binding to the Wuhan-Hu-1 RBD. Related to Figures 1D-E. **(A)** A previously described deep mutational scanning system was used to map mutations that escape antibody binding.[27] A library of yeast containing nearly all possible mutations to the Wu-Hu-1 RBD was incubated with antibody and fluorescence-activated cell sorting (FACS) was used to enrich for cells expressing RBD (detected with a C-terminal MYC tag, green star) with reduced antibody binding, detected using an anti-human IgG Fc-gamma secondary antibody. Deep sequencing was used to quantify the frequency of each mutation in the pre-selection and antibody-escape cell populations. We calculated each mutation's "escape fraction," the fraction of cells expressing RBD with that mutation that fell in the antibody escape FACS bin (ranging from 0 to 1). The site-level escape metric is the sum of the escape fractions of all mutations at a site. Experimental and computational filters were used to remove RBD mutants that were misfolded or unable to bind the ACE2 receptor as described.[25,27] **(B)** Antibody binding to the yeast-displayed unmutated Wu-Hu-1 RBD was measured by flow cytometry and Wu-Hu-1 RBD libraries were incubated with antibody at the IC90 ("1x antibody") for antibody-escape selections. **(C)** Representative plots of nested FACS gating strategy used to select for RBD+ single cells. Samples were gated by SSC-A versus FSC-A, SSC-W versus SSC-H, and FSC-W versus FSC-H) that also express RBD (FITC-A vs. FSC-A). (D) FACS gating strategy to select cells expressing RBD mutants with reduced antibody binding (cells in blue). Selection gates were set to exclude cells expressing unmutated Wu-Hu-1 RBD stained with 1x antibody (top row), and to capture up to 95% of cells expressing unmutated Wu-Hu-1 RBD stained with 0.1x antibody (second row from top). The FACS scatter plots are shown for the two library replicates. SSC-A, side scatter-area; FSC-A, forward scatter-area; SSC-W, side scatter-width; SSC-H, side scatter-height; FSC-W, forward scatter-width; FSC-H, forward scatter height; FITC-A, fluorescein isothiocyanate-area. (E) Mutation (top)- and site (bottom)-level correlations of escape scores between two independent biological replicate libraries. (F) The antibody-escape maps as shown in Figure 1D, but with mutations colored according to effect on RBD expression, as previously measured.[29]

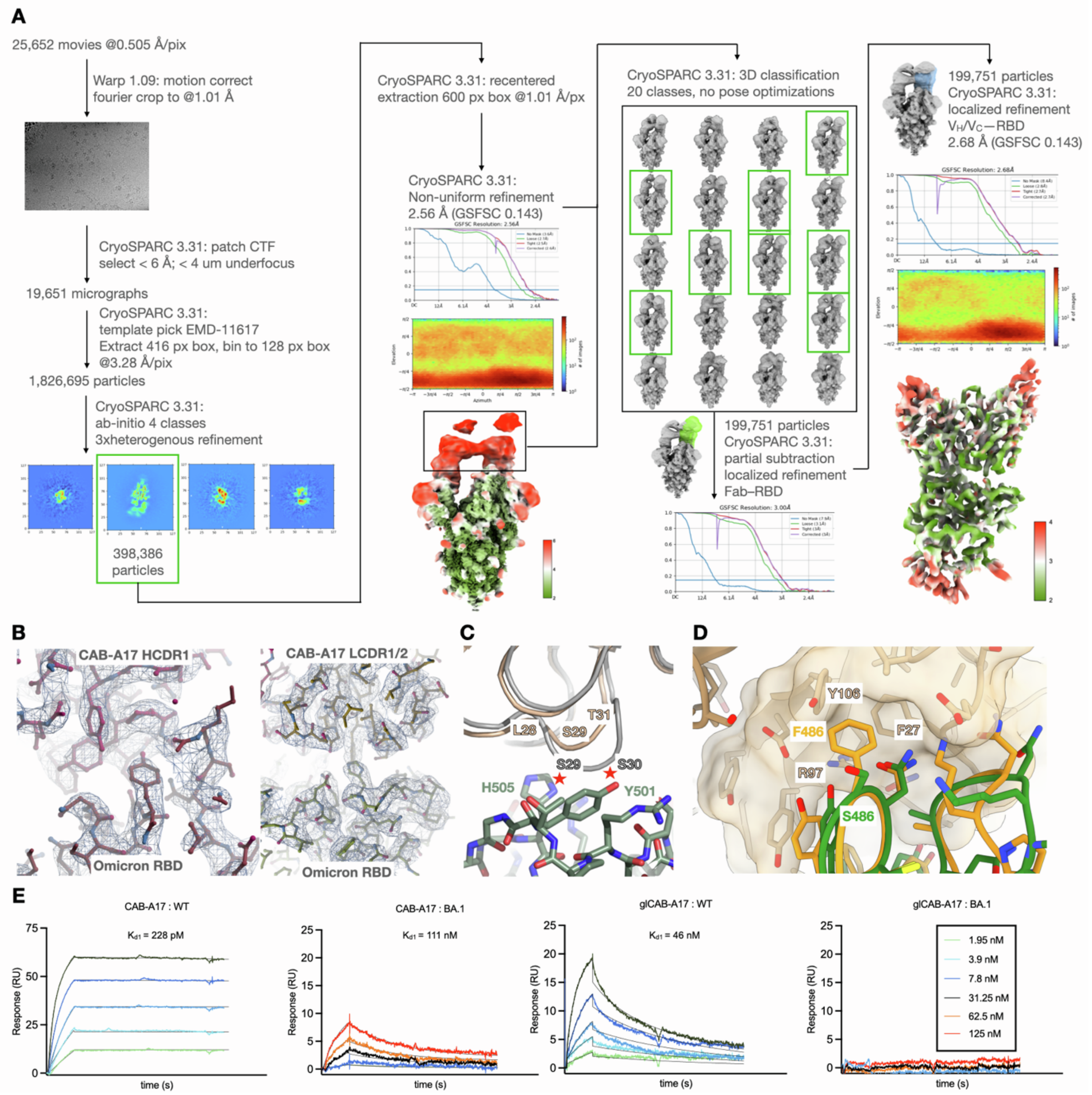

Figure S3. Cryo-EM processing workflow, light-chain shift and effects of F486S mutation. Related to Figures 2 and 3. **(A)** Cryo-EM processing workflow for the CAB-A17 Omicron spike complex. **(B)** Representative experimental map details. **(C)** The effect of the shortening of the CAB-A17 LCDR1 loop (CAB-A17 in light brown, C102 bound in grey). Potential sterical clashes denoted with a red star. **(D)** Difference in CAB-A17 interaction possibilities between BA.2.75 (orange-yellow;PDB:7YQY) and XBB (green; PDB:8IOU) due to an F486S mutation in XBB1. **(E)** Affinity of mature and germline-reverted CAB-A17 as measured using surface plasmon resonance (Biacore). Shown is the affinity of CAB-A17 IgG as well as its germline-reverted version (glCAB-A17) for immobilized RBD of the ancestral SARS-CoV-2 founder variant (WT) and Omicron BA.1 variant RBD (BA.1). Fitted (grey) is a bivalent binding model. K_{d1}, first dissociation rate constant. glCAB-A17 : BA.1 showed no affinity.

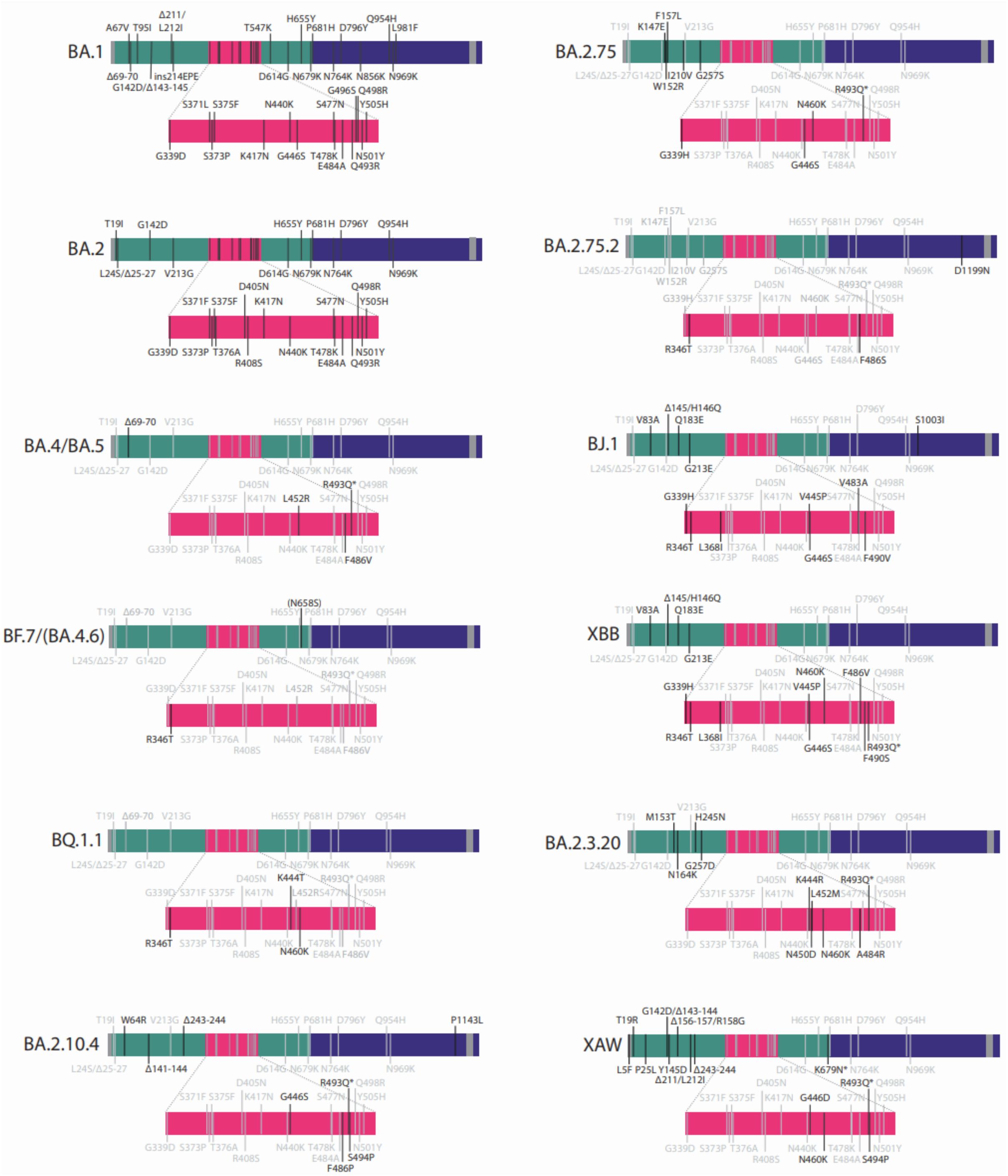

Figure S4. Schematic of the mutations in spike for the omicron sublineages forming the heterologous spike panel. Related to Figure 4.

Table S1. Cryo-EM data collection, refinement, and validation statistics. Related to Figure 2.

**Data collection**	Omicron spike + Fab CAB-A17 EMDB-16397 PDB 8C2R	Omicron + Fab CAB-A17 EMDB-42970 PDB 8V4F
Microscope	Krios G3i	-
Detector	K3 BioQuantum	-
Voltage (kV)	300	-
Electron exposure (e^{-}/Å^2)	54.6	-
Defocus range (μm)	–0.3 to –2.0	-
Frames	60	-
Magnification (nominal)	165,000	-
Final map pixel size (Å/px)	1.01	-
Symmetry imposed	C1	C1
No. of movies	25,652	-
No. of final particles images	398,386	199,751
Map resolution at 0.143 FSC threshold (Å)	2.56	2.68

Model refinement

Model composition

Non-hydrogen atoms	30,930	4,797
Protein residues	3,873	619
Non-protein residues	52 NAG	-

B-factors

Protein (Å^2)	80.7	84.9
Ligands (Å^2)	104	-

R.m.s. deviations

Bond lengths (Å)	0.007	0.002
Bond angles (°)	0.678	0.557

Validation[a]

MolProbity score	1.47	1.29
Clashscore	8.79	4.99
Poor rotamers (%)	0.00	0.19

Ramachandran plot		
Favored (%)	98.42	97.87
Allowed (%)	1.58	2.13
Outliers (%)	0.00	0.00

[a] All validations statistics according to MolProbity[52]

Table S2. Polar-interaction table CAB-A17 – Omicron (Omicron vs Wu-Hu-1 mutations in red). Related to Figure 2.

CAB-A17 HC	Atom	Distance (Å)	Atom	Omicron RBD
E26	O	3.0	ND2	N487
E26	OE2	3.8 (salt bridge)	NZ	K478
I28	N	3.5	O	A475
A31	O	3.1	OH	Y473
N32	ND2	3.1	O	A475
Y33	OH	2.6	O	L455
G54	N	2.6	OH	Y421
G54	O	3.4	ND2	N460
S56	OG	2.7	OD2	D420
S56	OG	3.8	OD1	N460
R97	NH1	3.0	OD1	N487
R97	NH2	2.9	OH	Y489

CAB-A17 LC	Atom	Distance (Å)	Atom	Omicron RBD
G92	O	3.1	NE2	H505

Table S3. Bad clashes according to Molprobity[52] in the modeled C102 binding to Omicron (Omicron vs Wu-Hu-1 mutations in red). Related to Figure 2.

C102 HC	Atom	Atom	Omicron RBD
N32	OD1	HB1	A475
G54	N	OH	Y421
G54	CA	OH	Y421
G54	H	HH	Y421
R94	NH2	OH	Y489
Y99	OH	OH	Y453
Y99	CE2	NH1	R493
Y99	HE2	NH1	R493
G54	N	OH	Y421
G54	O	ND2	N460
Y102	CZ	CE1	F486
Y102	CE2	HE1	F486
**C102 LC**	**Atom**	**Atom**	**Omicron RBD**
S29	HG	ND1	H505
S29	OG	ND1	H505
S29	CB	ND1	H505
S29	CB	CG	H505
S29	HG	CG	H505
S29	HB2	CG	H505
S29	HB2	CE1	H505
S29	HB2	CD2	H505
S29	HG	CE1	H505
S29	CB	CE1	H505
S29	CB	CB	H505
S29	OG	CE1	H505
S29	HB3	CB	H505
S30	HA	OH	Y501
S30	HA	CZ	Y501
S30	CA	CZ	Y501
S30	OG	HB2	S496
G92	O	NE2	H505
G92	O	CE1	H505
